# *Cryptosporidium parvum* Pyruvate Kinase Inhibitors With *in vivo* Anti-cryptosporidial Efficacy

**DOI:** 10.3389/fmicb.2021.800293

**Published:** 2022-01-03

**Authors:** Shahbaz M. Khan, Xuejin Zhang, William H. Witola

**Affiliations:** Department of Pathobiology, College of Veterinary Medicine, University of Illinois at Urbana-Champaign, Urbana, IL, United States

**Keywords:** *Cryptosporidium*, treatment, pyruvate kinase, inhibitors, *in vivo*

## Abstract

*Cryptosporidium parvum* is a highly prevalent protozoan parasite that causes a diarrheal disease in humans and animals worldwide. Thus far, the moderately effective nitazoxanide is the only drug approved by the United States Food and Drug Administration for treating cryptosporidiosis in immunocompetent humans. However, no effective drug exists for the severe disease seen in young children, immunocompromised individuals and neonatal livestock. *C. parvum* lacks the Krebs cycle and the oxidative phosphorylation steps, making it dependent solely on glycolysis for metabolic energy production. Within its glycolytic pathway, *C. parvum* possesses two unique enzymes, the bacterial-type lactate dehydrogenase (CpLDH) and the plant-like pyruvate kinase (CpPyK), that catalyze two sequential steps for generation of essential metabolic energy. We have previously reported that inhibitors of CpLDH are effective against *C. parvum*, both *in vitro* and *in vivo*. Herein, we developed an *in vitro* assay for the enzymatic activity of recombinant CpPyK protein and used it to screen a chemical compound library for inhibitors of CpPyK’s activity. The identified inhibitors were tested (at non-toxic concentrations) for efficacy against *C. parvum* using *in vitro* assays, and an *in vivo* mouse infection model. We identified six CpPyK inhibitors that blocked *in vitro* growth and proliferation of *C. parvum* at low micromolar concentrations (EC_50_ values ranging from 10.29 to 86.01 μM) that were non-toxic to host cells. Among those six compounds, two (NSC252172 and NSC234945) were found to be highly efficacious against cryptosporidiosis in immunocompromised mice at a dose of 10 mg/kg body weight, with very significant reduction in parasite load and amelioration of intestinal pathologies. Together, these findings have unveiled inhibitors for an essential molecular target in *C. parvum* and demonstrated their efficacy against the parasite *in vitro* and *in vivo*. These inhibitors are, therefore, potential lead-compounds for developing efficacious treatments for cryptosporidiosis.

## Introduction

Intracellular eukaryotic protozoan parasites belonging to the genus *Cryptosporidium* are members of the phylum Apicomplexa. Out of the currently documented 42 species of *Cryptosporidium* (Zahedi and Ryan, 2020), *Cryptosporidium parvum* is recognized as the major zoonotic species responsible for diarrheal infections in animals and humans worldwide (Ryan et al., 2014; Thomson et al., 2017). In particular, *C. parvum* is the most frequently identified enteric pathogen in pre-weaned calves (Cho et al., 2013), causing substantial morbidity resulting in weight loss and delayed growth (Thomson et al., 2017; Shaw et al., 2020). Among humans in developing countries, *Cryptosporidium* is the main cause of linear growth faltering and the second leading cause of moderate-to-severe diarrhea in infants (0–11 months of age) and a major cause of mortality and stunted growth in the second year of life (Kotloff et al., 2013; Nasrin et al., 2021). Cryptosporidiosis is regarded as a high-risk and often fatal opportunistic infection for immunocompromised patients such as those suffering from HIV/AIDS (O’Connor et al., 2011) or those receiving organ transplants (Danziger-Isakov, 2014; Bhadauria et al., 2015).

The life cycle of *C. parvum* consists of the asexual replication phase and the sexual reproduction phase leading to generation of infectious oocysts that persist in the environment (Fayer et al., 2000). While *C. parvum* has been recognized as an important etiological agent of diarrhea for over four decades (Navin and Juranek, 1984), neither fully effective therapeutic drugs nor prophylactic vaccines are currently available. Nitazoxanide, the only drug approved for treatment of cryptosporidiosis in immunocompetent human patients, is ineffective in the most susceptible populations including young children and immunocompromised individuals (Checkley et al., 2015). Further, there is no effective treatment available for *C. parvum* infections in cattle (Santin, 2020). Thus, there is an urgent need for the development of efficacious anti-cryptosporidial drugs.

Owing to the lack of genes encoding components of conventional apicomplexan drug targets such as the apicoplast, tricarboxylic acid cycle, and the adenosine triphosphate (ATP)-generating classical respiratory chain (Abrahamsen et al., 2004), drugs developed against various other apicomplexan parasites are ineffective against cryptosporidiosis. Nevertheless, the parasite possesses other unique pathways that are essential for its growth and replication within the host. Notably, *C. parvum* lacks a functional mitochondria, making it dependent on glycolysis for metabolic energy (Abrahamsen et al., 2004; Zhu, 2007). The glycolytic enzymes, therefore, are potential targets for developing therapeutics against *C. parvum*, provided that adequate parasite-versus-host selectivity is attained. Of interest are *C. parvum* pyruvate kinase (CpPyK), a plant-like pyruvate kinase enzyme, and *C. parvum* lactate dehydrogenase (CpLDH), a bacterial-type lactate dehydrogenase enzyme (Abrahamsen et al., 2004), that sequentially catalyze the last two glycolytic reactions to produce pyruvate and lactate, respectively, with generation of metabolic energy (ATP).

Previously, we have provided genetic evidence for the critical role of CpLDH in growth, infectivity, and multiplication of *C. parvum*, both *in vitro* and *in vivo* (Witola et al., 2017; Zhang et al., 2018). We have also identified CpLDH inhibitors that show antiparasitic activity against *C. parvum*, both *in vitro* and *in vivo* (Li et al., 2019). Herein, we undertook a chemical library screen to identify specific inhibitors for the enzymatic activity of recombinant CpPyK (rCpPyK) protein. Using mammalian cell culture infection assays and *in vivo* mouse infection models, we have demonstrated that some of those CpPyK inhibitors possess anti-cryptosporidial activity at low tolerable doses. Collectively, our results make a compelling case for further development of the identified glycolytic pathway inhibitors into the next generation of efficacious anti-cryptosporidial drugs.

## Materials and Methods

### *Cryptosporidium parvum* Propagation and Oocyst Purification

The AUCP-1 isolate of *C. parvum* was maintained and propagated by repeated passage in Holstein bull calves. Oocysts were purified from freshly collected calf feces by sequential sieve filtration, Sheather’s sugar flotation (Current, 2018), and discontinuous sucrose density gradient centrifugation (Arrowood and Sterling, 1987). Purified oocysts were washed and stored at 4°C in phosphate buffered saline (PBS) and used within 3 months of initial purification from feces, when viability remained above 75% as judged by microscopic evaluation of motile activity of excysted sporozoites. Sporozoites were excysted from *C. parvum* oocysts following a previously described procedure (Kuhlenschmidt et al., 2016). Briefly, about 1 × 10^8^ purified *C. parvum* oocysts were suspended in 500 μl of PBS and treated with an equal volume of 40% commercial laundry bleach for 10 min at 4°C. The oocysts were washed four times in PBS containing 1% (w/v) bovine serum albumin (BSA), resuspended in Hanks balanced salt solution (HBSS), and then incubated at 37°C for 60 min. An equal volume of warm 1.5% sodium taurocholate in HBSS was added to the oocysts followed by further incubation at 37°C for 60 min with occasional shaking. The excysted sporozoites were collected by centrifugation, washed in supplemented PBS, and resuspended in RPMI-1640 medium containing 10% fetal bovine serum (FBS). The sporozoites were separated from oocyst shells and unexcysted oocysts by filtering the suspension through a sterile 5 μm syringe filter (Millex™, Millipore). Purified sporozoites were enumerated with a hemocytometer and used immediately for the infection of cell monolayers.

### Cloning and Expression of rCpPyK Protein

cDNA was prepared from total RNA extracted from the AUCP-1 isolate of *C. parvum* and the CpPyK coding sequence (GenBank accession number XM_628040) was PCR-amplified from the cDNA using the primer pair 5′-*CTCGAG*ATGATTTCAAACGATCA-3′ (Forward, with the *Xho*I restriction site italicized and start codon in bold) and 5′-*GGATCC*TTAGGGGCACCTAACTAT-3′ (Reverse, with the *Bam*HI site italicized and stop codon in bold) for site-directed cloning at the *Xho*I/*Bam*HI site of the pET-15b expression vector (Novagen) in frame with the N-terminal hexahistidine tag (His-tag). The recombinant expression vector was sequenced to confirm identity, amplified in the K12 strain of *Escherichia coli* cells (NEB^®^ Turbo; New England Biolabs), and transformed into protein expression BL21-CodonPlus (DE3)-RIL *E. coli* (Agilent Technologies). Transformed *E. coli* were cultured at 37°C in Luria broth medium containing 100 μg/ml ampicillin and 34 μg/ml chloramphenicol to an absorbance of 0.6–0.8 at a wavelength of 600 nm, and protein expression was induced by addition of 1 mM isopropyl-β-D-thiogalactopyranoside. Bacterial cells were pelleted by centrifugation and resuspended in lysis buffer (50 mM NaH_2_PO_4_, 300 mM NaCl, and 10 mM imidazole, pH 8.0) containing a 1 × EDTA-free protease inhibitor cocktail, 600 units benzonase, and 30 kU lysozyme (EMD Millipore). The resuspended bacteria were lysed by sonication on ice and the cleared soluble fraction of the lysate was clarified by centrifugation at 13,000 rpm. The His-tagged recombinant protein was purified under native conditions by nickel-affinity chromatography according to the manufacturer’s instructions (Novagen). The wash buffer contained 50 mM NaH_2_PO_4_, 300 mM NaCl, and 20 mM imidazole, pH 8.0, while the elution buffer was composed of 50 mM NaH_2_PO_4_, 300 mM NaCl, and 250 mM imidazole, pH 8.0. An ultrafiltration centrifugal protein concentrator with a molecular weight cut-off of 30K (Thermo Scientific) was used to remove imidazole and concentrate the protein in dialysis buffer containing 5 mM Hepes–KOH (pH 7.8) and 0.5 mM DTT. The purity and concentration of the recombinant protein were evaluated by SDS/PAGE and a Qubit™ Protein Assay Kit (Life Technologies), respectively.

### CpPyK Enzyme Activity and Kinetics

**FIGURE 1:**
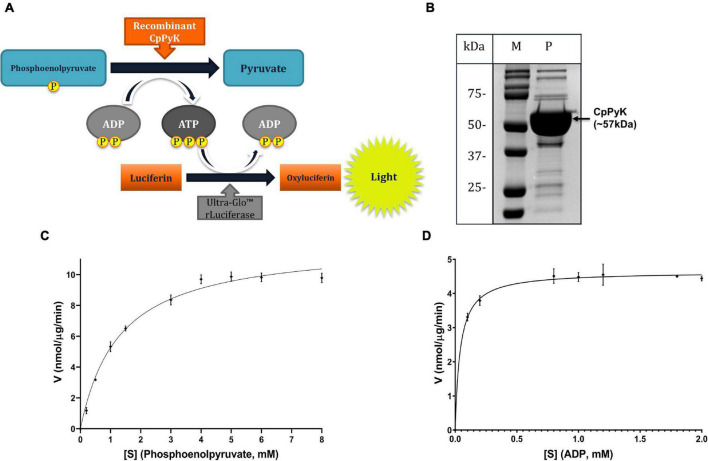
Analysis of the enzymatic activity of rCpPyK protein. **(A)** Illustration of the *in vitro* assay for the dephosphorylation of phosphoenolpyruvate to pyruvate using rCpPyK protein as the catalytic enzyme. The reaction is coupled to a luciferase assay involving the utilization of the generated ATP to phosphorylate luciferin to oxyluciferin whose luminescence is detected by a plate reader. **(B)** SDS-PAGE analysis of the nickel affinity column chromatography purified rCpPyK protein stained with Coomassie blue (Lane M: protein ladder; Lane P: CpPyK protein with the expected band size indicated at about 57 kDa). **(C,D)** Enzyme kinetics of rCpPyK on the substrate phosphoenolpyruvate and on the co-substrate ADP, respectively. The data shown represent the mean of three independent experiments with standard deviation (SD) error bars. ADP, Adenosine diphosphate; ATP, Adenosine triphosphate; CpPyK, *Cryptosporidium parvum* pyruvate kinase; V, the velocity of the reaction.

The *in vitro* enzymatic activity of the rCpPyK protein was determined by quantitating the amount of ATP produced by the transfer of a phosphate group from phosphoenolpyruvate to adenosine diphosphate (ADP) in the presence of varying concentrations of the recombinant protein (Figure 1A). A typical assay contained 0.5 mM phosphoenolpyruvate, 0.6 mM ADP, 50 mM Tris buffer pH 7.5, 60 mM MgSO_4_, 100 mM KCl, and varying concentrations of rCpPyK incubated for 3 h at room temperature. The ATP generated was detected in a reaction volume of 50 μl in white opaque-walled 96 well plates by the luciferase-based Cell Titer-Glo 2.0 reagent (Promega) following the manufacturer’s instructions. The enzyme kinetics of rCpPyK were determined by using varying phosphoenolpyruvate (0–8 mM) and ADP (0–2 mM) concentrations in an enzymatic reaction catalyzed by a fixed concentration of the recombinant protein (6 ng/μl). In all assays, reaction mixtures without rCpPyK were included as negative controls. Three independent assays were performed for each experiment, and samples were run in triplicate. The luminescence generated was recorded using a multi-mode microplate reader (Spectra Max iD3; Molecular Devices, United States). GraphPad PRISM^®^ v8 software was used to fit the Michaelis–Menten model directly to the substrate-velocity data to determine the enzymatic kinetic parameters for rCpPyK.

### Screening Compounds for Inhibitory Activity Against CpPyK

The Diversity Set VI chemical library (Supplementary Table 1) was obtained from the National Cancer Institute (NCI)/Developmental Therapeutics Program (DTP) Open Repository collection of chemical compounds.^1^ Compounds were individually reconstituted in molecular biology grade dimethyl sulfoxide (DMSO) from Sigma-Aldrich and stored at −20°C. From the library, 1,424 compounds were screened in 96-well plates using the rCpPyK catalyzed enzyme assay to identify potential CpPyK inhibitors. The reactions were performed in 50 μl reaction volume containing 0.5 mM phosphoenolpyruvate, 0.6 mM ADP, 50 mM Tris buffer pH 7.5, 60 mM MgSO_4_, 100 mM KCl, and 6 ng/μl of rCpPyK protein with or without compound. The concentration of DMSO in all reaction mixtures was 1% (v/v). Control reactions without rCpPyK protein were included for background subtraction. Following 3 h of incubation at room temperature, an equal volume of the Cell Titer-Glo 2.0 reagent was added to each well, and the luminescence produced was recorded as relative luminescence units (RLU) after 10 min of incubation using a multi-mode microplate reader (Spectra Max iD3; Molecular Devices, United States). The mean percent inhibition (MPI) of rCpPyK activity by each compound was derived by dividing the difference in luminescence (RLU) between the compound-treated wells and the DMSO-treated wells by the luminescence of the DMSO-treated wells and multiplying the product by 100:


(1) $$MPI=[\left(MeanRLU_{DMSO-treated}-MeanRLU_{Compound-treated}\right)÷MeanRLU_{DMSO-treated}]\times 100.$$

Half maximal inhibitory concentration (IC_50_) values were determined by applying a non-linear regression analysis curve fit to the mean dose-response data for varying concentrations of each compound using GraphPad PRISM^®^ v8. Reactions were performed in triplicate and repeated at least thrice.

### *In vitro* Compound Cytotoxicity Assays

Compounds were tested for cytotoxicity in human ileocecal colorectal adenocarcinoma cells [HCT-8 (HRT-18); ATCC^®^ CCL-244™, CVCL_2478] by using the cell proliferation reagent WST-1 (Roche) according to the manufacturer’s protocol. The WST-1 assay is a quantitative colorimetric assay for measurement of metabolically active cells. This assay is based on the reduction of the tetrazolium salt (WST-1) by viable cells. About 5 × 10^4^ HCT-8 cells were seeded per well in 96-well plates and grown overnight in 200 μl of RPMI-1640 medium (without phenol red) (Gibco) supplemented with 2.5 g/L of glucose, 1 mM sodium pyruvate, 1.5 g/L of sodium bicarbonate, 10% heat-inactivated FBS (Gibco), and 1 × Antibiotic-Antimycotic (Gibco) at 37°C with 5% CO_2_ in a humidified incubator. Upon reaching 80–90% confluency, cells were treated with the chemical compounds reconstituted in DMSO for 24 h. The volume of DMSO was kept below 1% of the total culture volume in all the wells to avoid DMSO cytotoxicity. Control wells received equivalent volumes of DMSO used in the reconstituted compounds. After 24 h of culture, 10 μl of the WST-1 reagent was added to each well, and the plates were incubated for 1 h at 37°C with 5% CO_2_ under dark conditions. The plates were shaken thoroughly and 150 μl of the medium from each well was transferred to a new clear flat-bottom black 96-well plate (Corning). Absorbance was read at a test wavelength of 440 nm and a reference wavelength of 690 nm using a multi-mode microplate reader (Spectra Max iD3; Molecular Devices, United States). The mean percent toxicity (MPT) of each compound was derived by dividing the difference in absorbance (OD) between the compound-treated cells and the DMSO-treated cells by the absorbance from the DMSO-treated cells and multiplying the product by 100:


(2) $$MPT=[\left(MeanOD_{DMSO-treated}-MeanOD_{Compound-treated}\right)÷MeanOD_{DMSO-treated}]\times 100.$$

The half maximal cytotoxic concentration (CC_50_) values were determined by using non-linear regression analysis in GraphPad PRISM^®^ v8. Assays were performed in triplicate and repeated three times.

### *In vitro* Testing of Anti-*Cryptosporidium* Activity of CpPyK Inhibitors

HCT-8 cells were grown to confluency in supplemented RPMI-1640 medium in 96-well plates. Once confluent, the cells were infected with 10^5^ freshly excysted *C. parvum* sporozoites per well, immediately followed by the addition of anti-CpPyK compounds (reconstituted in DMSO) to one set of wells. Control infected cells were treated with DMSO volumes equivalent to those used for the compound-treated cultures. Paromomycin reconstituted in distilled sterile water, served as a positive control at a concentration of 200 μM. The cell monolayers were processed by a direct immunofluorescence assay (Witola et al., 2017) after 48 h of culture at 37°C with 5% CO_2_. Briefly, the medium was removed from the culture wells, and the cells were washed two times with PBS before fixation with pre-chilled methanol-acetic acid (9:1) for 5 min at room temperature. Residual fixative was removed by rinsing the wells with PBS. Cells were rehydrated and permeabilized by washing twice with a buffer containing 0.1% Triton X-100, 0.35 M NaCl, and 0.13 M Tris-base, pH 7.6. Normal goat serum (5% in PBS) was used as a blocking agent, and the cell monolayer was stained with a fluorescein-labeled anti-*C. parvum* polyclonal antibody (Sporo-Glo™; Waterborne, Inc.) overnight at 4°C. The stained cells were washed twice with PBS followed by the addition of 200 μl water to each well. Plates were then imaged with an inverted fluorescence microscope using a 20× objective. The fluorescence generated by intracellular *C. parvum* parasites was quantified from nine microscopic fields per well of a 96-well plate using the batch process function in ImageJ version v1.50 (NIH, United States) after setting a threshold for the detection of parasites. Control wells with uninfected monolayers were included for background subtraction. Experiments were performed in triplicate and repeated at least three times.

Anti-*Cryptosporidium* half-maximal effective concentration (EC_50_) values of CpPyK inhibitors were determined by performing *in vitro C. parvum* infection assays as described above, with the exception that varying concentrations of compounds were used to treat infected HCT-8 cell cultures. One set of wells with confluent HCT-8 cells received CpPyK inhibitors immediately after *C. parvum* infection, while the same compounds were added to the other set of wells 2 h post-infection (PI). Control infected cells were treated with varying volumes of DMSO equivalent to the ones used for the compound-treated cultures. Cell monolayers were processed for immunofluorescence analysis after 48 h of incubation as described above. Samples were run in triplicate and three independent assays were performed. EC_50_ values were calculated using non-linear regression analysis of the mean dose-response curve data in GraphPad PRISM^®^ v8.

### *In vivo* Testing of the Anti-*Cryptosporidium* Efficacy of CpPyK Inhibitors

Eight weeks old male interferon-gamma knockout (IFN-γ KO) mice (B6.129S7-*Ifng^TM1Ts^*/J) were purchased from The Jackson Laboratory, United States, and allowed to quarantine and acclimatize for 1 week before the commencement of experiments. Before the start of the infection assays in mice, the tolerability of each inhibitor was determined by daily oral gavage with varying dosages (0–10 mg/kg mouse bodyweight) in groups of mice (*n* = 3 mice per group) for 8 days. During this period, mice were monitored daily for any changes in physical and mental activity, feeding, body weight, body temperature, fur condition, and body posture. The highest dose of each inhibitor that did not produce any signs of toxicity during the 8-day monitoring period was used as the maximum dose limit for subsequent *in vivo* infection studies. Mice were divided into infected and uninfected groups (*n* = 3 mice per group) and each individual mouse was housed in a separate cage lined with sterile gauze bedding. Each mouse from the infected group was infected by oral gavage administration of 10^4^ *C. parvum* AUCP-1 isolate oocysts suspended in 50 μl of PBS. Beginning day 3 PI, groups of mice were orally treated with CpPyK inhibitors (at the specified doses), sham (100 μl of 5% DMSO in PBS), or 1,000 mg/kg paromomycin (in sterile water) once daily for a total of 8 days. Fecal pellets were collected daily in individual sterile 15 ml tubes, and submerged in an equivalent volume of PBS containing a cocktail of penicillin (100 units/ml), streptomycin (100 μg/ml), chloramphenicol (34 μg/ml), and amphotericin (0.25 μg/ml), and stored at 4°C until use. Three independent replicate infection assays were performed. Mice were sacrificed at day 11 PI, and 5 cm of the distal small intestine just anterior to the cecum was resected and submerged in 10% neutral buffered formalin and submitted for histopathological processing in the Comparative Biosciences Histology Laboratory at the University of Illinois at Urbana-Champaign. Briefly, intestinal tissues preserved in 10% neutral buffered formalin were washed in 70% ethanol, embedded in paraffin, and sectioned transversely at a thickness of 5 μm. Sections were stained with hematoxylin and eosin. The slides were imaged using a Zeiss microscope fitted with a color camera.

### Quantitative Analysis of *Cryptosporidium parvum* Oocysts Load in Mice Feces

DNA was isolated from 250 mg of feces collected from individual mice using the QIAamp^®^ PowerFecal^®^ Pro DNA kit (Qiagen, United States) following the manufacturer’s protocol. Oocysts load per gram of feces was measured by quantitative real time PCR (qPCR) analysis of the Cp18s rRNA gene (GenBank accession number AF164102) using gene-specific primers: 5′-CTGCGAATGGCTCATTATAACA-3′ (Forward) and 5′-AGGCCAATACCCTACCGTCT-3′ (Reverse), described previously (Parr et al., 2007). To generate a quantification standard curve, fecal samples were obtained from uninfected mice and spiked with 10^8^ *C. parvum* oocysts per gram of feces, followed by isolation of DNA as described above. Ten-fold serial dilutions of the extracted DNA were made and used as quantification standards for qPCR. *C. parvum* oocysts load quantification for the test mice was performed using DNA samples from the infected feces. Each 20 μl qPCR reaction contained 10 μl of PowerUp™ SYBR™ Green Master Mix (Applied Biosystems, United States), 500 nM of each primer, and 2 μl of DNA template. After 2 min of initial denaturation at 95°C, 40 cycles of denaturation at 95°C for 15 sec and annealing/extension at 60°C for 1 min were performed in a 7500 Real-Time PCR System (Applied Biosystems, United States). The oocyst load per gram of feces was derived by the 7500-system software using the generated quantification standard curves (Supplementary Figure 1).

### Statistical Analysis

Statistical analyses were done by two-way analysis of variance (ANOVA) with the Tukey’s multiple comparison *post hoc* test using GraphPad PRISM^®^ v8. *P*-values of 0.05 or less were considered significant.

### Ethics

Animal study protocols were approved by the University of Illinois Institutional Animal Care and Use Committee under protocol numbers 21091 and 20036 for the use of Holstein calves and mice respectively. All experiments involving the use of animals were carried out in strict compliance with the recommendations and guidelines in the United States Department of Agriculture Animal Welfare Act and the National Institute of Health Public Health Service Policy on the Humane Care and Use of Animals. All efforts were made to minimize the pain and suffering of animals.

## Results

### Enzymatic Activity and Kinetics of rCpPyK Protein

The 1,581 bp long open reading frame of the CpPyK gene translates into a 526 amino acid protein with an estimated molecular weight of 56.4 kDa and an isoelectric point of 6.78.^2^ We sequenced the cloned CpPyK coding sequence amplified from *C. parvum* cDNA and observed a 100% homology when aligned with the nucleotide sequence reported in genome databases (CryptoDB gene ID: cgd1_2040; GenBank accession number: XM_628040).

To analyze the activity of the CpPyK protein in catalyzing the transfer of a phosphate group from phosphoenolpyruvate to ADP, we expressed CpPyK as a His-tagged protein and nickel affinity column chromatography-purified it in its native form. The purified rCpPyK protein containing the vector-derived His-tag was of the expected molecular size of approximately 57 kDa (Figure 1B). Using the ATP detection-based CpPyK enzymatic assay (Figure 1A), we found that rCpPyK protein depicted concentration- and pH-dependent catalytic activity with optimal activity at 6 ng/μl and pH 7.5, respectively. The catalytic activity of the recombinant protein was consistent with Michaelis–Menten kinetics on the substrate (phosphoenolpyruvate) and the co-substrate (ADP) (Figures 1C,D), with kinetic parameters (*K*_*m*_ and *V*_*max*_) that were comparable to those previously reported for CpPyK (Table 1; Denton et al., 1996).

**TABLE 1:** rCpPyK enzyme kinetic parameters on substrates.

Parameter	Phosphoenolpyruvate	ADP
*V*_*max*_ (nmol/μg/min)	12.05 (6.82^a^)	4.64
*K*_*m*_ (mM)	1.29 (0.32*^a^*)	0.04

*^a^Reported by Denton et al. (1996).*

### Identification of Non-toxic CpPyK Inhibitors

**FIGURE 2:**
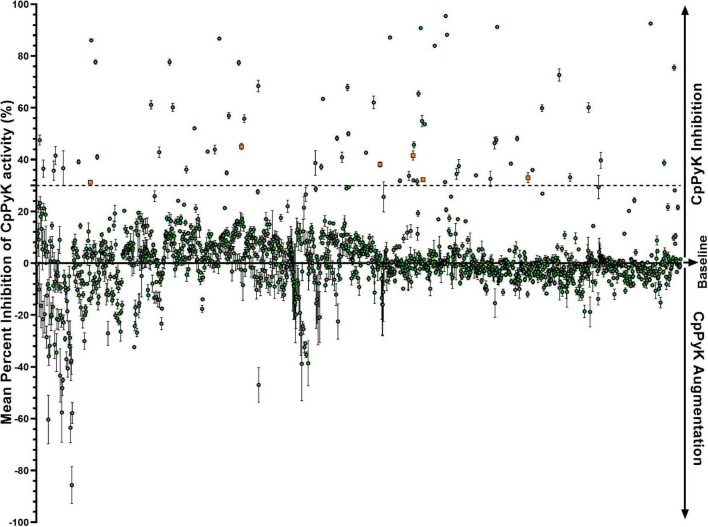
Effect of compounds from the NCI Diversity Set VI chemical library on the catalytic activity of rCpPyK protein. Individually reconstituted compounds were used at a final concentration of 50 μM in the CpPyK catalyzed reaction for the transfer of a phosphate group from phosphoenolpyruvate to ADP, yielding pyruvate and ATP. The mean percent inhibition of rCpPyK activity by each compound was derived by dividing the difference in luminescence between the compound-treated wells and the DMSO-treated wells by the luminescence of the DMSO-treated wells and multiplying the product by 100. The baseline mean percent inhibition of 0 was for the reaction without compound, but with an equivalent volume of DMSO used to reconstitute the compounds. Compounds with mean percent inhibition values greater than 0 were designated as inhibitors of the activity of rCpPyK, while those with mean percent inhibition values less than 0 were classified as augmenters. The orange squares indicate the six top hits found to have significant inhibitory effect on the *in vitro* growth of *C. parvum* as shown in Figure 4. Each reaction was performed in triplicate, and the data shown represent the mean of three independent experiments. Bars represent standard errors of the mean (SEM).

To identify selective inhibitors for the rCpPyK catalytic activity, we screened a library consisting of 1,424 chemical compounds using the *in vitro* CpPyK enzymatic assay. We identified 70 compounds that displayed substantial *in vitro* inhibition (>30%) of the rCpPyK catalytic activity at a concentration of 50 μM (Figure 2). In addition, there were several other compounds that exhibited low inhibitory activity (<30%) against rCpPyK, but for logistical reasons were not pursued further in order to limit the number of compounds that were subsequently investigated in detail. The remaining compounds either had no effect or augmented the activity of rCpPyK and were also not analyzed further.

**FIGURE 3:**
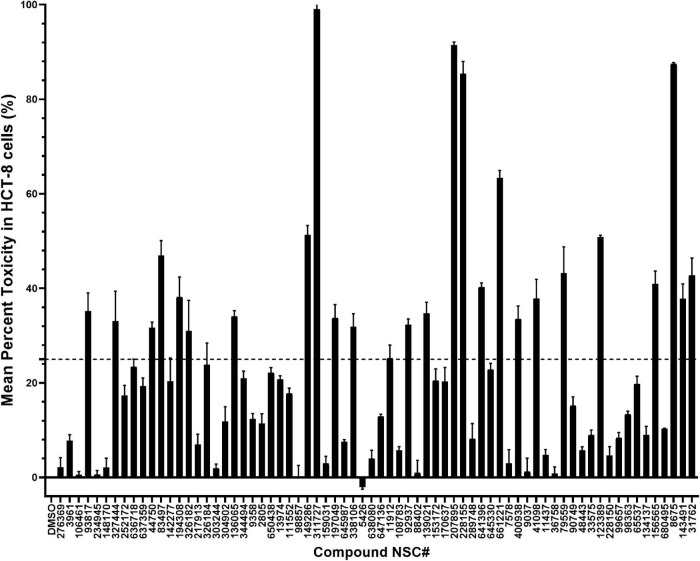
Mean percent toxicity (MPT) values of test compounds in HCT-8 cells. Individually reconstituted compounds were incubated with uninfected HCT-8 cells for 24 h at a final concentration of 50 μM. Control wells without chemical compound, but in which equivalent volumes of DMSO (chemical compound solvent) were added were also set up. Following 24 h of culture, a colorimetric assay using the cell proliferation reagent WST-1 was used for the quantification of cell viability. The MPT value of each compound was derived by dividing the difference in absorbance between the compound-treated cells and the DMSO-treated cells by the absorbance from the DMSO- treated cells and multiplying the product by 100. The baseline MPT of 0 was for the reaction without compound, but with an equivalent volume of DMSO used to reconstitute the compounds. Each reaction was performed in triplicate, and the data shown represent the mean of three independent experiments. Bars represent standard errors of the mean (SEM).

Prior to the evaluation of rCpPyK inhibitors for *in vitro* anti-cryptosporidial effect, we tested them for cytotoxicity against the host cell line (HCT-8) that was used for *in vitro* culture of *C. parvum*. The cytotoxic effect of compounds in HCT-8 cells was evaluated by quantifying the cleavage of the tetrazolium salt (WST-1) to formazan by metabolically active cells. In the initial cytotoxicity screen, of the 70 rCpPyK inhibitors, 44 depicted low cytotoxicity (<25%) after 24 h of treatment of the cell cultures with the inhibitors at a concentration of 50 μM, and those compounds were, therefore, selected for *in vitro* efficacy studies (Figure 3). On the other hand, the remaining 26 compounds were found to inhibit the host cells’ viability by more than 25% and were thus not pursued further due to potential toxicity issues.

### rCpPyK Inhibitors With *in vitro* Anti-*Cryptosporidium* Efficacy

**FIGURE 4:**
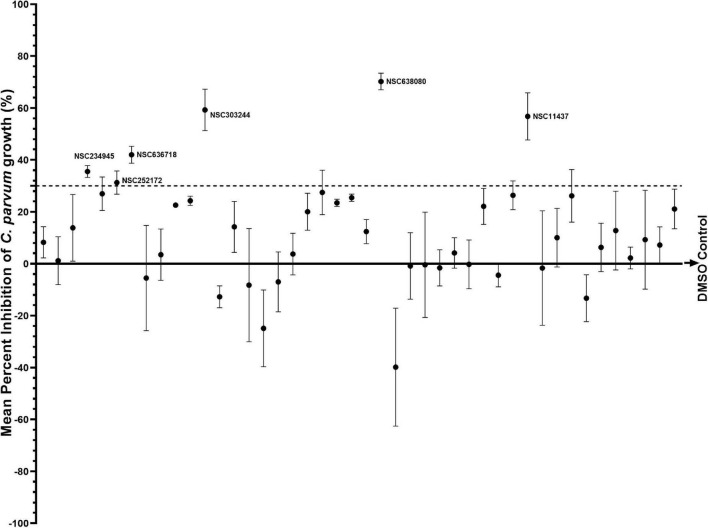
Analysis of the effect of rCpPyK inhibitors on the *in vitro* growth of *C. parvum* in HCT-8 cells. Equal amounts of freshly excysted sporozoites of *C. parvum* were inoculated into HCT-8 cells in culture and compounds at 25 μM concentration were added immediately after infection. Control infected cells were treated with volumes of DMSO equivalent to those used in the compound-treated cultures. Paromomycin reconstituted in distilled sterile water was added to a separate set of wells as a positive control at 200 μM final concentration. The cultures were analyzed for parasite infectivity and proliferation by an immunofluorescence assay after 48 h of incubation. The fluorescence generated by intracellular *C. parvum* parasites was quantified and used to calculate the mean percent parasite inhibition values for each compound. The data shown represent the mean of three independent experiments. Bars represent standard errors of the mean (SEM).

To determine the effect of rCpPyK inhibitors on the intracellular growth and replication of *C. parvum*, we performed *in vitro* infection and treatment assays using HCT-8 cell monolayers to culture *C. parvum*. rCpPyK inhibitors that were found to be non-toxic to host cells at 50 μM were screened initially at 25 μM (half the concentration used for primary cytotoxicity screening) to evaluate their *in vitro* efficacy against *C. parvum*. All experimental data were normalized with DMSO-treated wells and paromomycin-treated (Marshall and Flanigan, 1992) wells that represented negative and positive treatment controls, respectively. Among the 44 compounds tested, a total of six compounds (NSC234945, NSC252172, NSC636718, NSC303244, NSC638080, and NSC11437) exhibited significant inhibitory effect on the *in vitro* proliferation and viability of *C. parvum* when compared to the untreated parasites after 48 h of culture (Figure 4). We selected those compounds for secondary analysis using varying concentrations of each compound at different time-points of infection to determine their *in vitro* anti-cryptosporidial EC_50_ values. To start with, the selected compounds were analyzed at varying concentrations (ranging from 0 to 1,000 μM) for *in vitro* cytotoxicity against uninfected HCT-8 cells using the WST-1 cell proliferation assay, and the half maximal cytotoxicity concentration (CC_50_) values derived (Table 2). As a guide, for each compound, the highest concentration that was tested for anti-cryptosporidial activity did not exceed 50% of its CC_50_ value.

**TABLE 2:** Enzyme inhibition, cytotoxicity, *in vitro* efficacy, and selectivity index values of selected compounds.

Compound	Enzyme inhibition IC_50_ (μM)	HCT-8 CC_50_ (μM)	Anti-*C. parvum* EC_50_ (μM)		Selectivity index (CC_50_/EC_50_)
			0 h PI	2 h PI	
NSC234945	234.9	432.1	86.01	89.58	5.02
NSC252172	123.3	266.5	17.61	18.73	15.10
NSC636718	116.7	106.5	33.78	33.56	3.15
NSC303244	242.5	660.2	10.29	15.79	64.15
NSC638080	97.14	749.6	10.87	14.68	68.96
NSC11437	156.1	612.5	23.80	27.27	25.73

**FIGURE 5:**
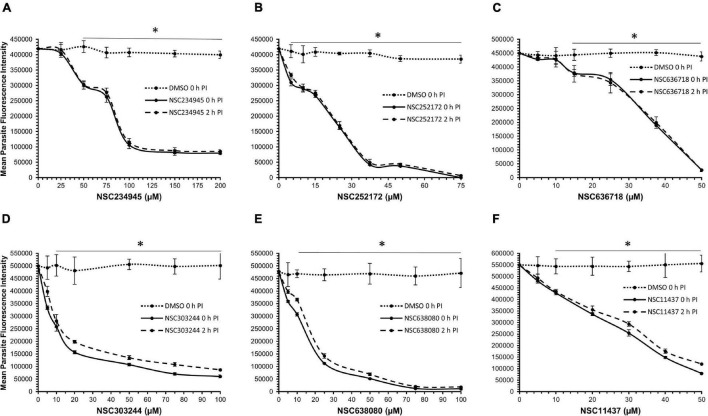
Effect of varying concentrations of rCpPyK inhibitors on the growth of *C. parvum* in HCT-8 cells. Equal amounts of freshly excysted sporozoites of *C. parvum* were inoculated into HCT-8 cells in culture and varying concentrations of **(A)** NSC234945, **(B)** NSC252172, **(C)** NSC636718, **(D)** NSC303244, **(E)** NSC638080, and **(F)** NSC11437 were added at the time of infection (solid line) or added 2 h post-infection (PI) (dashed line). Control infected cells (dotted line) were treated immediately PI with volumes of DMSO equivalent to those used in the compound-treated cultures. After 48 h, the cultures were analyzed for parasite proliferation by immunofluorescence assays. The fluorescence generated by intracellular *C. parvum* merozoites was quantified and is shown on the *Y*-axis representing the parasite load. The data shown represent the mean of three independent experiments. Bars represent standard errors of the mean (SEM) with level of statistical significance as compared to the DMSO-treated controls indicated by asterisk (*P < 0.05).

To determine the compounds’ concentration-dependent effect against *C*. *parvum in vitro*, each compound was tested by adding it to the HCT-8 cells culture shortly before or 2 h after infection with *C*. *parvum* sporozoites in order to assess the effect of the compound on host cell invasion by the parasites, and the effect of the compound on intracellular parasites, respectively. When the cultures were analyzed by an immunofluorescence assay after 48 h PI, all six compounds (NSC234945, NSC252172, NSC636718, NSC303244, NSC638080, and NSC11437) showed a significant (*P* < 0.05) concentration-dependent inhibitory effect on the proliferation of intracellular *C. parvum* parasites in HCT-8 cells in comparison with the control infected cultures without compound treatment (Figures 5A–F). The treatment of infected HCT-8 cultures resulted in concentration-dependent decreases in parasite viability regardless of whether the compound (NSC234945, NSC252172, NSC636718, and NSC11437) treatment commenced at 0 h PI or 2 h PI (without any notable significant difference between the two time points of treatment). However, compounds NSC303244 and NSC638080 showed higher potency when treatment commenced at 0 h PI than at 2 h PI, suggesting that exposure of the sporozoites to the compounds prior to host cell invasion reduced their viability and led to a reduction in the number of parasites that invaded the cells. The EC_50_ values derived from the dose-response curves for the compounds, when compared to their CC_50_ values, showed that compounds NSC638080 and NSC303244 had the best SIs (Table 2), suggesting that they had good safety margin with regard to toxicity in mammalian cells, but with best potencies at low EC_50_ concentrations against *C. parvum*. Compounds NSC636718 and NSC234945 had the least SIs, suggesting a narrower safety margin (Table 2).

### rCpPyK Inhibitors With *in vivo* Efficacy Against *Cryptosporidium parvum* Infection

Based on the *in vitro* infection and treatment assay results described above, we selected NSC638080 and NSC303244 for *in vivo* testing, because of their high SIs that implied wider safety margins in terms of toxicity to mammalian cells. While NSC252172 had a comparatively lower SI, it was also selected for *in vivo* testing because of its relatively high *in vitro* potency against *C. parvum* (Table 2). Additionally, even though NSC234945 had comparatively lower SI, it was also selected for *in vivo* testing because of its high solubility in DMSO, which facilitated its use at high doses reconstituted in relatively small volumes of DMSO. NSC11437 was not pursued further because of its low solubility and difficulties in sourcing sufficient amounts for *in vivo* use. Additionally, NSC636718 was also not pursued further because it had the lowest SI and low *in vitro* anti-cryptosporidial efficacy in comparison to other compounds.

**FIGURE 6:**
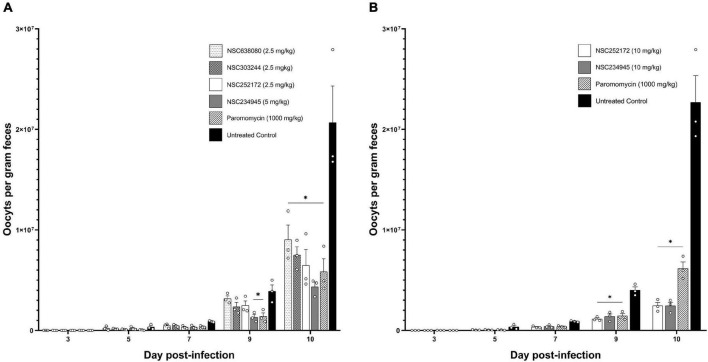
Real-time PCR quantification of the load of *C. parvum* oocysts in fecal samples of infected mice treated with or without rCpPyK inhibitors. Male IFN-γ KO mice (three animals per group) were infected with 10^4^ *C. parvum* oocysts and from the third day post-infection, daily treatment was commenced with **(A)** 2.5 mg/kg NSC638080, 2.5 mg/kg NSC303244, 2.5 mg/kg NSC252172, or 5 mg/kg NSC234945, and **(B)** 10 mg/kg NSC252172 or 10 mg/kg NSC234945. In both experiments **(A,B)** paromomycin at 1,000 mg/kg was used as positive control treatment, while the untreated control group of mice was administered an equivalent volume of the solvent used to reconstitute the test compounds (5% DMSO in water). Oocyst shedding per gram of feces was measured by qPCR of the *C. parvum* 18S rRNA gene, and the equivalent oocysts per gram feces were derived using a standard curve. The data shown represent the means for fecal oocysts load from three mice per group. Bars represent standard errors of the mean (SEM) with level of statistical significance as compared to the untreated control mice indicated by asterisk (*P < 0.05).

Prior to use in mice, the targeted highest dose of 10 mg/kg was tested for tolerability in uninfected mice. For the selected four compounds (NSC638080, NSC303244, NSC252172, and NSC234945) the dose of 10 mg/kg daily oral administration did not induce any toxicity signs (changes from normal physical activity, respiration, body temperature, feeding pattern, body posture, fur condition, or occurrence of death) over 8 days of treatment. However, for the initial evaluation of the compounds’ anti-*Cryptosporidium* efficacy in mice, a lower dose of 2.5 mg/kg was used for NSC638080, NSC303244, and NSC252172, while 5 mg/kg of NSC234945 was used. This was because, compared to the other three compounds, NSC234945 had depicted an *in vitro* EC_50_ concentration that was 2–3-fold higher, suggesting lower potency. Paromomycin was used as a positive control drug at 1,000 mg/kg once daily orally (Griffiths et al., 1998). The load of *C*. *parvum* oocysts shed in mice feces was determined by using real time PCR quantification of the *C*. *parvum* 18s rRNA gene. As expected, all the infected mice started shedding detectable levels of *C. parvum* oocysts in their feces by day 3 PI, indicating that at the time point (day 3 PI) when treatment commenced, the infection was patent in all the infected mice. From day 5 PI onward, there were significantly progressive increases in oocysts load in the feces of the untreated mice, peaking at about 2.1 × 10^7^ oocysts per gram feces on day 10 PI (Figure 6A). By day 8 PI, all the mice in the untreated infected group had become visibly sick, showing signs of progressive disease including rough hair coat, hunched back, reluctance to move, and weight loss. In comparison, from day 5 PI onward, mice in the groups treated with test compounds or paromomycin all maintained lower loads of oocysts in their feces when compared to the untreated mice (Figure 6A). By day 9 PI, there was a notable difference in oocysts load among the different compound treatments, with NSC234945 and paromomycin depicting significantly (*P* < 0.05) lower oocysts loads than the other treatments and the untreated group (Figure 6A). By day 10 PI (when the oocyst load peaked) compounds treatment groups had 2–6-fold lower oocysts loads than the untreated group, with compound NSC234945 showing the highest potency, followed by paromomycin, NSC25172, NSC303244, and NSC638080, in that order (Figure 6A). Consistent with the lower oocysts counts, when compared to the untreated, mice from all the treated groups depicted relatively normal physical parameters including activity, posture, and appetite. Because the untreated mice became moribundly ill by day 10 PI, for ethical considerations, all mice were sacrificed on day 11 PI and intestinal samples submitted for histopathology.

Because NSC234945 and NSC252172 showed higher potency than the rest of the compounds, we tested them further at a higher dose of 10 mg/kg to determine if they would depict dose-dependent efficacy. Intriguingly, this increase in dose led to about threefold reduction in oocysts load by day 9 PI when compared to the untreated mice (Figure 6B). And by day 10 PI, both NSC234945 and NSC252172 had about ninefold lower oocysts load than the untreated mice (Figure 6B), indicating that higher dose increased the compounds’ *in vivo* efficacy against *C. parvum*. Noteworthy, the higher dose (10 mg/kg) of NSC234945 and NSC252172 showed about 2.5-fold higher potency than paromomycin (1,000 mg/kg) by day 10 PI (Figure 6B). These mice were also sacrificed on day 11 PI and the distal small intestines submitted for histopathology.

**FIGURE 7:**
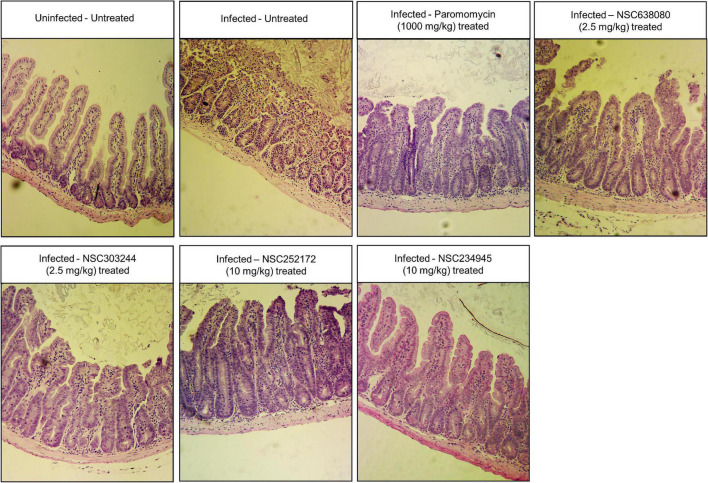
Histological analysis of the distal small intestines of mice infected with *Cryptosporidium parvum* with or without treatment. Male IFN-γ KO mice (*n* = 3 per group) were infected with 10^4^ *C. parvum* oocysts, and from the third day post-infection, daily oral treatment was commenced with NSC638080 (2.5 mg/kg), NSC303244 (2.5 mg/kg), NSC252172 (10 mg/kg), NSC234945 (10 mg/kg), or paromomycin (1,000 mg/kg). The untreated-uninfected control group of mice was administered an equivalent volume of the solvent used to reconstitute the test compounds (5% DMSO in water). After day 11 post-infection, mice were sacrificed, and the distal small intestinal tissue processed for histology and stained with hematoxylin and eosin. The uninfected-untreated control mice’s samples depicted intact intestinal epithelium with prominent villi. The infected–untreated mice depicted denuded villi, infiltration of inflammatory cells and hypertrophy of crypts. In contrast, samples from infected mice treated with the test compounds or paromomycin had notably reduced pathological changes (intact intestinal epithelium with prominent villi), especially treatment with compounds NSC252172 and NSC234945. The images are representative of samples analyzed from three mice per treatment group.

*Cryptosporidium parvum* colonizes mostly the distal small intestines, causing villous atrophy, erosion and ulceration of the intestinal mucosa. As such, we performed histopathological examination of sections of the small intestines resected from the distal part of the small intestines. As expected, while uninfected mice had normal intestinal mucosa, infected untreated mice had microscopic lesions characterized by severe villous atrophy, mucosal erosion, hypertrophy of the crypts of Lieberkuhn, and infiltration of inflammatory cells (Figure 7). In contrast, treatment of infected mice with the compounds evidently ameliorated the effects of *C. parvum* infection in the intestinal mucosa, with NSC234945 showing the best efficacy, followed by NSC252172 (Figure 7). These findings were consistent with the significantly lower oocysts loads attributed to NSC234945 and NSC252172 treatments. Notably, NSC303244 and NSC638080, though having lower efficacy than NSC234945 and NSC252172, still reduced intestinal pathology in a comparative manner to paromomycin (Figure 7).

## Discussion

An ideal approach to developing effective and safe drugs against parasites is through targeting the disruption of the activity of the parasite’s unique molecules that are essential for its survival. The complete and annotated genome sequence of *C. parvum* indicates that, while the parasite lacks genes for conventional molecular drug targets found in other important protozoan parasites, it has several genes encoding unique plant-like and bacterial-like enzymes that catalyze potentially essential biosynthetic and metabolic pathways (Abrahamsen et al., 2004). Importantly, *C. parvum* lacks the tricarboxylic acid cycle and the oxidative phosphorylation steps for generation of metabolic energy (ATP) (Abrahamsen et al., 2004), making the parasite solely dependent on anaerobic respiration (glycolytic pathway) for the generation of ATP which is critical for its survival and pathogenesis in the host (Witola et al., 2017; Zhang et al., 2018). Previous studies have highlighted the importance of glycolytic enzymes such as glucose-6-phosphate isomerase (CpGPI) and hexokinase (CpHK) as potential drug targets for the treatment of cryptosporidiosis (Eltahan et al., 2018, 2019). Furthermore, we have found that inhibitors for *C. parvum*’s unique bacterial-type lactate dehydrogenase (CpLDH) in the parasite’s glycolytic pathway can stop growth of the parasite and prevent disease in infected mice models (Li et al., 2019). Herein, we targeted the *C. parvum* pyruvate kinase (CpPyK) enzyme that catalyzes the step immediately upstream of the CpLDH catalytic step. CpPyK serves as the key metabolic control in *C. parvum*’s glycolytic cycle, in that it catalyzes the irreversible conversion of phosphoenolpyruvate to pyruvate and generates ATP (Berg et al., 2002).

We cloned and sequenced the coding sequence of CpPyK from *C. parvum* cDNA and found it to have significantly low homology to mammalian pyruvate kinases, consistent with the previous reports that it differs both functionally and structurally from its mammalian counterparts (Denton et al., 1996; Cook et al., 2012). We established an *in vitro* enzymatic assay in which we used natively purified recombinant CpPyK as the enzyme to catalyze the dephosphorylation of phosphoenolpyruvate to pyruvate and production of ATP. Using this assay, we validated that CpPyK follows Michaelis–Menten saturation kinetics, consistent with previous observations (Denton et al., 1996). By using this CpPyK enzyme-based *in vitro* assay, we screened a chemical compound library and identified specific inhibitors for the catalytic activity of CpPyK, suggesting that those compounds could also potentially block the activity of the *bona fide* CpPyK in the parasites, leading to loss of metabolic energy for the parasite. Given that mammalian cells possess pyruvate kinases, we first determined the CpPyK inhibitors’ cytotoxicity (CC_50_) concentrations in order to guide our selection of the concentrations for the *in vitro* anti-cryptosporidial assays. From these assays we identified CpPyK inhibitors (NSC234945, NSC252172, NSC636718, NSC303244, NSC638080, and NSC11437) that had no toxicity to mammalian cells at their effective concentrations against the activity of CpPyK. These findings corroborated reports that CpPyK has distinct functional as well as structural properties when compared to mammalian pyruvate kinases, suggesting that CpPyK inhibitors may not affect mammalian pyruvate kinases because of their divergent structural differences (Fothergill-Gilmore and Michels, 1993; Entrala and Mascaró, 1997). Additionally, mammals seem to be less sensitive to the inhibition of glycolysis because, unlike *C. parvum*, they possess multiple pyruvate kinase isoforms, a fully functional Krebs cycle, and the ability to use alternative energy sources including amino acids and fatty acids (Fothergill-Gilmore and Michels, 1993).

We then determined the *in vitro* anti-cryptosporidial efficacy of the candidate compounds in infected HCT-8 monolayers and identified compounds (NSC234945, NSC252172, NSC636718, NSC303244, NSC638080, and NSC11437) that had concentration-dependent inhibitory effect against the growth and proliferation of *C. parvum* parasites at low micromolar concentrations that were not toxic to mammalian host cells. Notably, the SIs of the six test compounds ranged from 3.2 to 69.0, indicating that they were all efficacious against *C. parvum* at concentrations that were tolerable to host mammalian cells (Kaminsky et al., 1996). The infection-treatment assays we used involved infecting HCT-8 cells with excysted *C*. *parvum* sporozoites that infect host cells and transform into proliferative merozoites, a process that requires metabolic energy. Therefore, by inhibiting the activity of CpPyK, the inhibitors blocked the key enzyme for the generation of metabolic energy, thereby curtailing the growth and replication of intracellular *C. parvum*. Paromomycin, which was used as a positive control drug in this study, has a reported *in vitro* anti-*Cryptosporidium* EC_50_ of 450 μM (Li et al., 2019). Comparatively, the six compounds that showed *in vitro* efficacy against *C. parvum* all had much lower EC_50_ values (ranging from 10.29 to 86.01 μM), suggesting better efficacy than paromomycin.

The IFN-γ KO mouse, which is highly susceptible to *C. parvum* infection, is a widely used acute infection model for evaluating compounds for *in vivo* anti-*Cryptosporidium* efficacy. Typically, *C. parvum*-infected IFN-γ KO mice develop gastrointestinal disease, characterized by extensive lower intestinal epithelial cells infection and severe mucosal damage, with associated clinical signs including depression, anorexia, weight loss, and death within 2–4 weeks (Griffiths et al., 1998). Treatment of *C. parvum*-infected IFN-γ KO mice with paromomycin has been shown to significantly limit parasite load and clinical disease, and to prevent death (Griffiths et al., 1998). Therefore, we used the IFN-γ KO mice to test the CpPyK inhibitors for *in vivo* efficacy against *C. parvum*. We found that treatment of the infected mice with compounds NSC252172, NSC303244, NSC638080 (at dose of 2.5 mg/kg), or NSC234945 (at a dose of 5 mg/kg) led to a significant reduction in the load of *C. parvum* oocysts shed in mice’s feces, and prevented manifestation of clinical disease and intestinal pathology in a manner that was comparable to treatment with paromomycin at 1,000 mg/kg. Comparatively, NSC252172 and NSC234945 depicted better efficacy, while NSC638080 had the least efficacy. Interestingly, at a higher dose of 10 mg/kg (that was tolerable in mice), both NSC252172 and NSC234945 showed significantly better efficacy than paromomycin at 1,000 mg/kg. These findings corroborated the *in vitro* efficacy results, thus positioning CpPyK inhibitors as promising lead-compounds for the development of efficacious anti-cryptosporidial drugs.

Lipinski’s and Veber’s rules describe a set of key physicochemical properties that determine the probability of drug candidates to be orally bioavailable in humans (Lipinski et al., 2001; Veber et al., 2002). The chemical structures of the CpPyK inhibitors (NSC234945, NSC252172, NSC636718, NSC303244, NSC638080, and NSC11437) with anti-cryptosporidial efficacy all conform to the Lipinski’s and Veber’s rules for the accepted criteria for drug-likeness, including their molecular masses, hydrophobicity, hydrogen bond donors/acceptors, rotatable bonds, and polarity, as listed in Supplementary Table 2. *Cryptosporidium* spp. are minimally invasive, and their development is restricted to an unusual intracellular but extra-cytoplasmic location in the host epithelial cells (Guérin and Striepen, 2020). Therefore, an orally active anti-cryptosporidial drug must effectively penetrate both host and parasite membranes to reach its intended target in the parasite. It is well known that octanol-water partition coefficients, cLog(*P*), provide a good estimate of a compound’s lipophilicity, which is a good measure of its ability to penetrate cellular membranes, including gastrointestinal absorption (Artursson and Karlsson, 1991). Generally, more lipophilic drugs tend to diffuse faster into the lipid cell membranes, suggesting that the relatively high cLog(*P*) value of 4.4 for NSC252172 (Supplementary Table 2) entailed enhanced lipophilicity, and thus likely favored its bioavailability *in vivo*, making it reach its intended molecular target in substantial amounts. However, too high cLog(*P*) may be counterproductive as it can negatively impact aqueous solubility and target molecule binding. Thus, the relatively lower cLog(*P*) value of 1.2 for compound NSC234945 would tend to maintain its aqueous solubility for optimized drug-likeness and anti-parasitic potency. Additionally, a molecule’s topological polar surface area (Å) value that is lower than 80 tends to improve the drug-likeness for a molecule. Interestingly, all the compounds we found to have anti-cryptosporidial efficacy had Å values that were significantly lower than 80 (Supplementary Table 2).

Structure-wise, the diversity of the aromatic/heteroaromatic portion among the CpPyK inhibitors extends to dihydroquinazoline (NSC303244), dihydronaphthalenone (NSC252172), and 3,5-dipyridyl-triazole (NSC234945) skeletons (Supplementary Figure 2). It is noteworthy that the aforementioned structural motifs are very often found in various approved medications and biologically active natural products, e.g., anthraquinone (Malik and Müller, 2016), and others representing the so-called “privileged scaffolds” often utilized in library design and drug discovery (Welsch et al., 2010; Alagarsamy et al., 2018; Aggarwal and Sumran, 2020). Most importantly, these scaffolds are highly amenable to structural modification for the synthesis of derivatives with enhanced potency and safety. In conclusion, the impressive anti-cryptosporidial efficacy and safety, both *in vitro* and *in vivo*, for the CpPyK inhibitors identified in the current study provide excellent starting compounds for the development of the much-needed novel anti-cryptosporidial therapeutics for both humans and animals.

## Data Availability Statement

The original contributions presented in the study are included in the article/Supplementary Material, further inquiries can be directed to the corresponding author.

## Ethics Statement

Animal study protocols were approved by the University of Illinois Institutional Animal Care and Use Committee under protocol numbers 21091 and 20036 for the use of Holstein calves and mice respectively. All experiments involving the use of animals were carried out in strict compliance with the recommendations and guidelines in the United States Department of Agriculture Animal Welfare Act and the National Institute of Health Public Health Service Policy on the Humane Care and Use of Animals. All efforts were made to minimize the pain and suffering of animals.

## Author Contributions

WW conceptualized and designed the study. SK conducted most of the experiments with some help from XZ and WW. SK and WW analyzed the data, constructed the figures and tables, and wrote the manuscript. WW supervised the project and edited and corrected the manuscript. All authors were involved in interpretation of the data and approved the final manuscript for submission.

## Conflict of Interest

The authors declare that the research was conducted in the absence of any commercial or financial relationships that could be construed as a potential conflict of interest.

## Publisher’s Note

All claims expressed in this article are solely those of the authors and do not necessarily represent those of their affiliated organizations, or those of the publisher, the editors and the reviewers. Any product that may be evaluated in this article, or claim that may be made by its manufacturer, is not guaranteed or endorsed by the publisher.

## Footnotes

1
http://dtp.cancer.gov

2
http://cryptodb.org

## Supplementary Material

Supplementary Figure 1 A representative standard curve generated by the 7500-system software for quantification of *C. parvum* oocyst load in mice fecal samples.

Supplementary Figure 2 Chemical structures of the inhibitors for CpPyK enzyme that have anti-cryptosporidial efficacy.
