# Supplementary figures and images for: Cryptosporidium parvum Pyruvate Kinase Inhibitors With in vivo Anti-cryptosporidial Efficacy
Source: Front Microbiol. 2022 Jan 3;12:800293. doi: 10.3389/fmicb.2021.800293 (PMC8761912; doi:10.3389/fmicb.2021.800293)

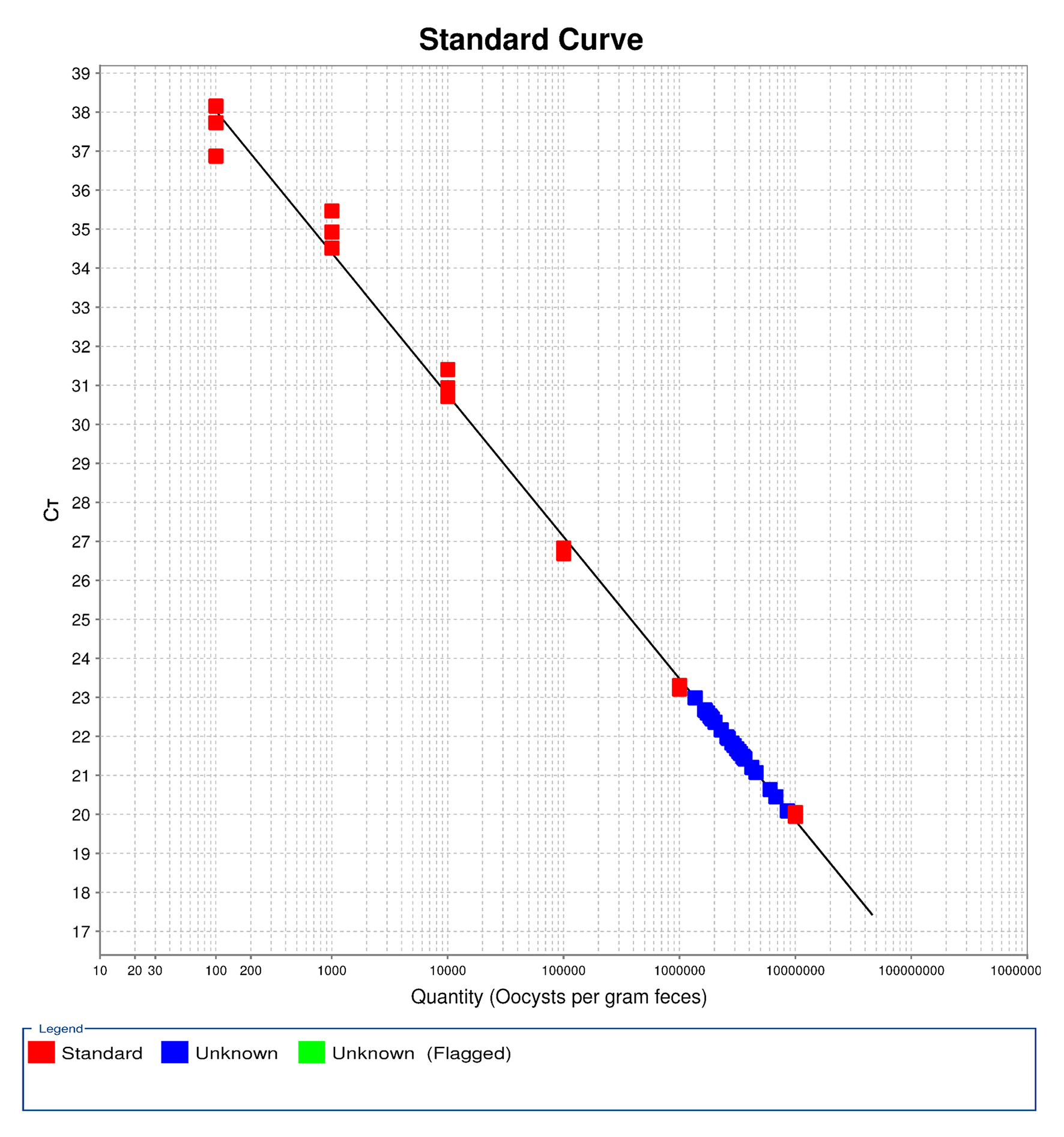

Supplement: Supplementary Figure 1 — A representative standard curve generated by the 7500-system software for quantification of C. parvum oocyst load in mice fecal samples. [file Image_1.TIF]

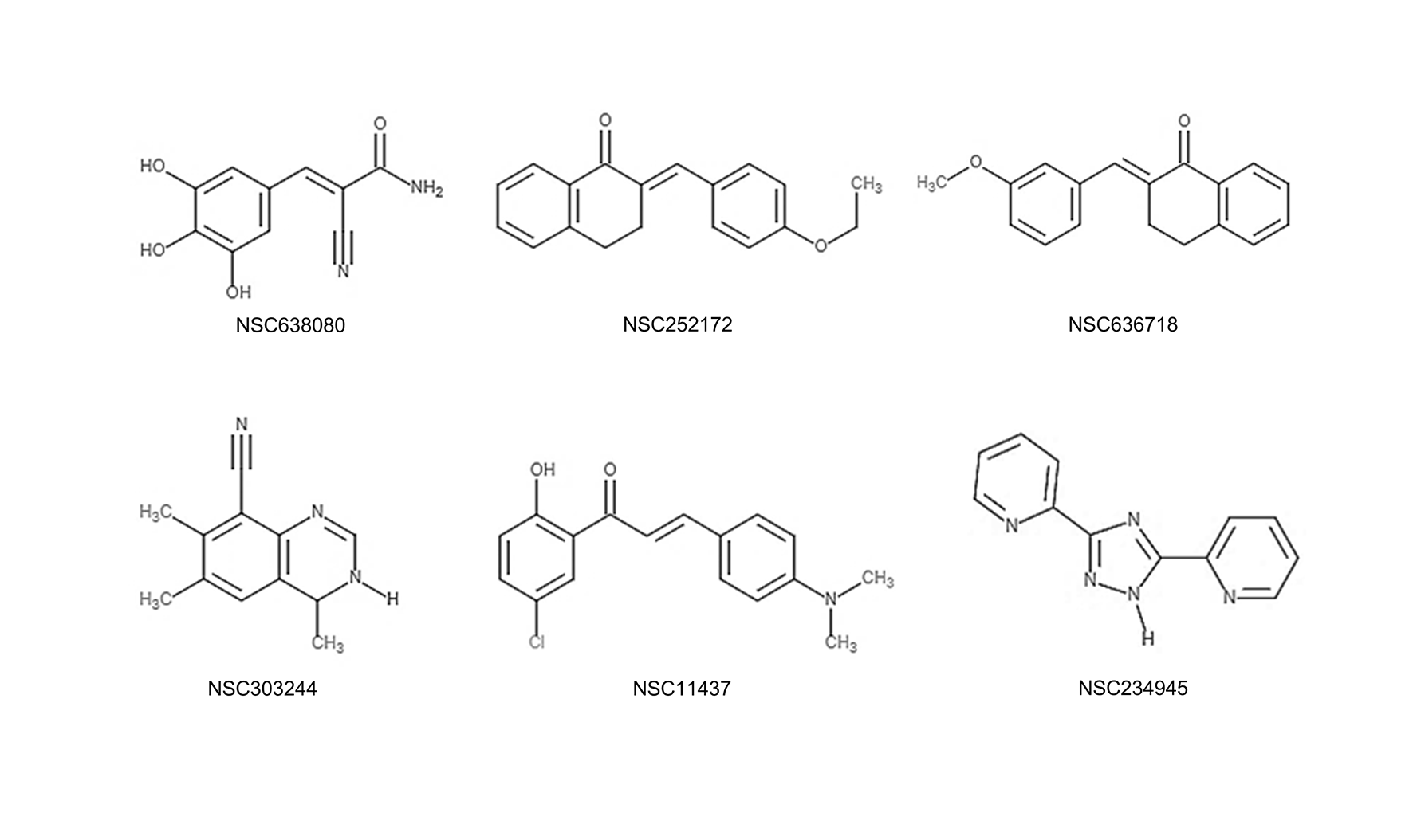

Supplement: Supplementary Figure 2 — Chemical structures of the inhibitors for CpPyK enzyme that have anti-cryptosporidial efficacy. [file Image_2.TIF]
