# Supplementary material for: Cryptosporidium parvum Pyruvate Kinase Inhibitors With in vivo Anti-cryptosporidial Efficacy
Source: Front Microbiol. 2022 Jan 3;12:800293. doi: 10.3389/fmicb.2021.800293 (PMC8761912; doi:10.3389/fmicb.2021.800293)
Supplement: Supplementary file 3 [file Table_1.PDF]

Supplementary Table S1. Diversity Set VI chemical compounds.

Plate Number	Well ID	NSC Number	Molecular Weight	Molecular Formula
4862	A02	109885	181	C7H11N5O
4862	B02	111107	184	C8H12N2OS
4862	C02	116565	199	C6H5N3O3S
4862	D02	117386	170	C5H6N4O3
4862	E02	118832	191	C10H9NOS
4862	F02	119969	188	C11H12N2O
4862	G02	120312	196	C10H16N2O2
4862	H02	122131	173	C10H7NO2
4862	A03	123458	193	C14H11N
4862	B03	125197	193	C8H11N5O
4862	C03	127216	162	C3H6N4S2
4862	D03	127458	195	C12H9N3
4862	E03	127947	199	C10H17NO3
4862	F03	128068	183	C12H9NO
4862	G03	131982	194	C10H14N2S
4862	H03	134577	164	C9H12N2O
4862	A04	134580	167	C8H9NO3
4862	B04	134784	182	C10H14O3
4862	C04	134785	197	C11H19NO2
4862	D04	135351	151	C9H13NO
4862	E04	136065	191	C10H13N3O
4862	F04	145180	167	C5H5N5S
4862	G04	147829	186	C11H10N2O
4862	H04	150982	188	C10H8N2O2
4862	A05	151901	189	C11H11NO2
4862	B05	152632	174	C6H10N2O2S
4862	C05	154316	153	C4H3N5O2
4862	D05	154718	183	C11H9N3
4862	E05	155196	169	C10H7N3
4862	F05	155698	200	C11H8N2S
4862	G05	155703	196	C11H8N4
4862	H05	156571	171	C9H17NO2
4862	A06	160005	168	C9H16N2O
4862	B06	162292	196	C11H20N2O
4862	C06	162915	197	C10H7N5
4862	D06	163104	198	C9H14N2OS
4862	E06	163158	165	C7H7N3O2
4862	F06	163920	184	C11H8N2O

4862	G06	164965	187	C7H7ClN2S
4862	H06	166900	156	C6H8N2O3
4862	A07	169458	185	C8H11NO2S
4862	B07	169566	151	C6H5N3S
4862	C07	173101	193	C9H11N3O2
4862	D07	176324	195	C5H7ClN2O4
4862	E07	177952	156	C4H4N4O3
4862	F07	191029	183	C11H21NO
4862	G07	194242	161	C8H7N3O
4862	H07	194243	176	C9H12N4
4862	A08	195031	185.31	C11H23NO
4862	B08	203065	191	C13H21N
4862	C08	206630	172	C8H16N2O2
4862	D08	227309	189	C11H11NO2
4862	E08	234764	196	C8H8N2O4
4862	F08	246415	200	C11H8N2S
4862	G08	269905	188	C11H12N2O
4862	H08	272275	159	C11H13N
4862	A09	276369	182	C8H10N2O3
4862	B09	278741	177	C9H11N3O
4862	C09	279834	187	C11H13N3
4862	D09	284701	152	C9H16N2
4862	E09	287065	178	C7H6N4O2
4862	F09	287495	182	C8H10N2O3
4862	G09	288686	189	C10H5ClN2
4862	H09	295701	189	C9H7N3S
4862	A10	303244	199	C12H13N3
4862	B10	303603	200	C7H8N2O5
4862	C10	303800	171	C7H9NO4
4862	D10	304902	186	C12H14N2
4862	E10	311723	189	C9H7N3S
4862	F10	321484	165	C7H7N3S
4862	G10	331198	155	C6H9N3S
4862	H10	331208	175	C8H9N5
4862	A11	335649	166	C5H6N6O
4862	B11	338205	175	C5H3ClN2O3
4862	C11	339578	199	C10H9N5
4862	D11	341902	178	C9H10N2O2
4862	E11	342460	183	C7H9N3O3
4862	F11	344494	175	C6H4Cl2N2
4862	G11	351110	183	C8H9NO4

4862	H11	361056	198	C7H6N2O3S
4863	A02	366808	197	C7H7N3S2
4863	B02	370387	161	C6H3N5O
4863	C02	372063	191	C10H9NO3
4863	D02	407282	190.24	C11H14N2O
4863	E02	513815	178	C7H6N4S
4863	F02	650438	179	C9H9NO3
4863	G02	664971	169	C8H15N3O
4863	H02	672441	158	C8H18N2O
4863	A03	1451	239.28	C13H13N5
4863	B03	1620	215.21	C10H9N5O
4863	C03	1751	224	C8H16O7
4863	D03	2561	240	C12H16O5
4863	E03	2805	246.26	C14H14O4
4863	F03	3001	214	C11H18O4
4863	G03	4263	225	C12H7N3O2
4863	H03	4921	214	C10H6N4O2
4863	A04	5995	213	C12H11N3O
4863	B04	6866	232	C10H11Cl2NO
4863	C04	6910	250	C13H18N2O3
4863	D04	8090	247	C17H13NO
4863	E04	8179	203	C9H9N5O
4863	F04	8481	208	C12H16O3
4863	G04	8813	238	C10H14N4O3
4863	H04	9064	204	C9H16O5
4863	A05	9341	237	C12H15NO4
4863	B05	9358	226	C13H14N4
4863	C05	9461	202	C6H4BrNO2
4863	D05	10091	240	C14H12N2O2
4863	E05	10416	243	C18H13N
4863	F05	10428	204	C12H12O3
4863	G05	10995	241	C10H9BrO2
4863	H05	11149	235	C13H11ClO2
4863	A06	11150	235	C13H11ClO2
4863	B06	11826	228	C10H8N6O
4863	C06	11891	225	C9H7NO2S2
4863	D06	11991	226	C14H14N2O
4863	E06	13653	218	C12H10O4
4863	F06	13974	247	C16H13N3
4863	G06	14304	222	C10H14N4O2
4863	H06	14540	219	C8H14NO4P

4863	A07	15362	227	C13H13N3O
4863	B07	15364	242	C13H14N4O
4863	C07	15372	235	C16H13NO
4863	D07	15571	246	C7H7AsO5
4863	E07	16416	234	C14H18O3
4863	F07	16813	216	C15H20O
4863	G07	17129	242	C10H14N2O3S
4863	H07	19063	230	C6H6N4S3
4863	A08	19096	222	C10H14N4O2
4863	B08	19108	244	C11H8N4OS
4863	C08	19115	217	C11H15N5
4863	D08	19487	220	C10H12N4S
4863	E08	19637	226	C13H10N2O2
4863	F08	20045	205	C13H19NO
4863	G08	21034	237	C10H7NO6
4863	H08	21678	232	C10H12N6O
4863	A09	22939	224	C12H20N2O2
4863	B09	23123	240	C14H12N2O2
4863	C09	23247	237	C13H11N5
4863	D09	23248	227	C13H13N3O
4863	E09	23672	222	C10H8ClN3O
4863	F09	23895	221	C12H15NO3
4863	G09	23906	201	C8H13ClN4
4863	H09	24035	227	C13H13N3O
4863	A10	25368	216	C11H20O4
4863	B10	26744	238	C15H10O3
4863	C10	27032	223	C15H13NO
4863	D10	27628	208	C9H12N4S
4863	E10	28341	210	C12H10N4
4863	F10	29471	213	C10H13ClN2O
4863	G10	29620	212	C11H20N2O2
4863	H10	112975	191	C11H13NO2
4863	A11	173103	196	C6H4N4O2S
4863	B11	281639	156	C5H8N4O2
4863	C11	365560	187	C7H9NOS2
4863	D11	3961	228	C11H8N4S
4863	E11	9852	240	C14H12N2O2
4863	F11	14303	222	C10H14N4O2
4863	G11	21725	244	C15H20N2O
4863	H11	17339	213	C14H15NO
4864	A02	31208	248	C12H12N2O2S

4864	B02	31664	233	C6H8AsNO4
4864	C02	31741	203	C5H6AsNO3
4864	D02	32838	202	C11H10N2O2
4864	E02	33005	243	C13H9NO2S
4864	F02	34488	239	C13H13N5
4864	G02	34769	218	C7H8ClN3O3
4864	H02	34774	230	C8H10N2O4S
4864	A03	34777	214	C8H10N2O5
4864	B03	35676	220.18	C11H8O5
4864	C03	35964	238	C11H14N2O4
4864	D03	36425	241	C11H19N3OS
4864	E03	36520	238	C10H14N4O3
4864	F03	36582	219	C12H13NO3
4864	G03	37003	244	C9H12N2O4S
4864	H03	37812	212	C12H8N2S
4864	A04	38042	237	C12H15NO4
4864	B04	38490	249	C12H11NO3S
4864	C04	38743	248	C12H12N2O4
4864	D04	38845	242	C12H10N4S
4864	E04	38983	224	C12H16O4
4864	F04	39336	223	C9H13N5S
4864	G04	40467	224	C10H8S3
4864	H04	40500	237	C12H15NO4
4864	A05	40614	224	C15H12O2
4864	B05	40669	209	C8H11N5S
4864	C05	41092	215	C11H9N3O2
4864	D05	42014	228	C9H12N2O3S
4864	E05	44688	232	C12H12N2O3
4864	F05	45153	222	C9H10N4OS
4864	G05	45291	214	C12H10N2S
4864	H05	46615	233	C11H15N5O
4864	A06	47617	248	C9H12O8
4864	B06	47619	216	C11H20O4
4864	C06	48964	210	C14H14N2
4864	D06	49652	225	C14H11NO2
4864	E06	49701	230	C12H14N4O
4864	F06	50633	237	C12H15NO4
4864	G06	51331	238	C14H10N2S
4864	H06	51936	214	C10H18N2O3
4864	A07	55770	206	C11H8ClNO
4864	B07	56455	235	C10H13N5S

4864	C07	57103	235	C9H5N3O5
4864	D07	57165	202	C11H10N2S
4864	E07	57318	225	C12H19NO3
4864	F07	57345	226	C13H22O3
4864	G07	57794	219	C10H13N5O
4864	H07	58907	237	C10H15N5S
4864	A08	59776	220	C15H12N2
4864	B08	60034	244.29	C14H16N2O2
4864	C08	60266	228	C9H12N2O3S
4864	D08	60419	203	C15H9N
4864	E08	61888	207	C15H13N
4864	F08	61910	231	C12H13N3O2
4864	G08	62665	205	C5H2Cl2N4O
4864	H08	63001	236	C15H12N2O
4864	A09	63865	244	C10H16N2O3S
4864	B09	63963	248	C12H16N2.C2H4O2
4864	C09	66837	230	C9H14N2O3S
4864	D09	67546	203	C9H17NO4
4864	E09	69421	250	C16H14N2O
4864	F09	70534	240	C13H12N4O
4864	G09	73170	244	C10H10ClNO4
4864	H09	75241	247	C8H11BrN2O2
4864	A10	75885	234	C12H18N4O
4864	B10	77596	244	C10H14ClN3O2
4864	C10	78130	243	C12H13N5O
4864	D10	79139	246	C17H14N2
4864	E10	79253	244	C13H12N2O3
4864	F10	79538	205	C10H11N3O2
4864	G10	80141	250	C13H9Cl2N
4864	H10	81018	242	C10H14N2O3S
4864	A11	81120	249.33	C16H11NS
4864	B11	81213	237	C6H5ClN2O4S
4864	C11	81660	217	C9H15NO3S
4864	D11	81703	224	C15H16N2
4864	E11	83715	247	C16H13N3
4864	F11	85179	220	C10H12ClF2N
4864	G11	85326	211	C8H13N5O2
4864	H11	87352	212	C10H12O3S
4865	A02	87822	244	C10H17N2O3P
4865	B02	88811	212	C13H12N2O
4865	C02	88883	215	C12H13N3O

4865	D02	88962	210	C10H10O5
4865	E02	88998	236	C10H15Cl2NO
4865	F02	89249	224	C10H12N2O4
4865	G02	89258	218	C16H10O
4865	H02	89723	232	C8H12N2O4S
4865	A03	91516	212	C12H8N2O2
4865	B03	92207	208	C9H5FN2O3
4865	C03	92794	245.24	C12H11N3O3
4865	D03	93817	227	C13H9NOS
4865	E03	96491	241	C12H23N3O2
4865	F03	98683	211	C6H5N5O4
4865	G03	98857	211	C13H9NS
4865	H03	99796	230	C12H6O5
4865	A04	100120	241	C12H11N5O
4865	B04	101266	219	C12H17N3O
4865	C04	101777	250	C8H15N2O5P
4865	D04	102086	237	C10H11N3O2S
4865	E04	102288	205	C11H15N3O
4865	F04	103770	213	C5H3N5OS2
4865	G04	103775	228	C5H4N6OS2
4865	H04	106208	248	C11H10ClN5
4865	A05	106282	249	C17H15NO
4865	B05	106461	240	C12H8N4S
4865	C05	106506	224	C14H12N2O
4865	D05	106570	238.29	C14H14N4
4865	E05	106863	242	C13H10N2O3
4865	F05	108235	229	C11H11N5O
4865	G05	108750	250	C16H14N2O
4865	H05	108753	209	C13H11N3
4865	A06	108972	223	C10H9NO3S
4865	B06	109084	214	C13H14N2O
4865	C06	109086	228	C14H16N2O
4865	D06	109466	245	C14H15NO3
4865	E06	109719	204	C10H6ClN3
4865	F06	111552	202	C12H10O3
4865	G06	112677	240	C15H16N2O
4865	H06	114490	226	C14H14N2O
4865	A07	114831	243	C10H13NO6
4865	B07	117554	250	C18H18O
4865	C07	117741	217	C10H7N3O3
4865	D07	117922	210	C7H6N4O4

4865	E07	118723	239	C15H17N3
4865	F07	120286	220	C9H8N4OS
4865	G07	120307	227	C11H9N5O
4865	H07	120844	210	C12H22N2O
4865	A08	121781	240	C14H12N2O2
4865	B08	122280	204	C10H8N2OS
4865	C08	122297	239	C10H13N3O2S
4865	D08	122376	226	C15H14O2
4865	E08	122987	212	C14H16N2
4865	F08	123141	214	C8H10N2O3S
4865	G08	124146	245	C13H15N3O2
4865	H08	125043	212	C12H12N4
4865	A09	125727	226	C10H14N2O2S
4865	B09	126405	232	C5H2Cl4N2
4865	C09	126757	220.19	C8H8N6O2
4865	D09	128141	236	C13H20N2O2
4865	E09	128737	210	C12H10N4
4865	F09	128751	229	C13H9ClN2
4865	G09	129260	241	C9H8FN3S2
4865	H09	130872	236	C15H12N2O
4865	A10	40383	226	C11H10N6
4865	B10	42846	213	C13H15N3
4865	C10	53710	204	C8H12O6
4865	D10	57670	230	C14H6N4
4865	E10	62611	212	C12H8N2S
4865	F10	68982	231	C8H8Cl2N4
4865	G10	70959	201	C6H5ClN4O2
4865	H10	71795	246	C17H14N2
4865	A11	101653	209	C10H15N3O2
4865	B11	109174	241.25	C13H11N3O2
4865	C11	42212	223	C14H9NO2
4865	D11	50405	202	C3H6O4S3
4865	E11	50572	248	C12H16N4S
4865	F11	56906	214	C7H10N4O2S
4865	G11	113486	209	C9H15N5O
4865	H11	73054	234	C14H10N4
4866	A02	131986	208	C11H16N2S
4866	B02	133195	229	C8H11N3O3S
4866	C02	133356	214	C8H6N8
4866	D02	139257	235	C10H9N3S2
4866	E02	140892	244	C13H12N2OS

4866	F02	143348	243	C13H13N3S
4866	G02	144958	242	C13H10N2O3
4866	H02	144982	226	C12H18O4
4866	A03	149046	228	C10H10ClNO3
4866	B03	149286	236.27	C15H12N2O
4866	C03	150954	231	C14H17NS
4866	D03	152551	236	C15H12N2O
4866	E03	153330	220	C11H12N2OS
4866	F03	153365	249	C8H15N3O6
4866	G03	153399	237	C15H11NO2
4866	H03	154295	231	C13H13NO3
4866	A04	156616	215	C5H5N5O3S
4866	B04	157767	211	C9H9NO5
4866	C04	157940	250	C15H22O3
4866	D04	159031	223	C15H13NO
4866	E04	163144	211	C12H9N3O
4866	F04	164208	220	C13H20N2O
4866	G04	164511	206	C12H18N2O
4866	H04	164678	203	C11H13N3O
4866	A05	165883	227	C14H13NO2
4866	B05	166583	219	C6H4Cl2N4O
4866	C05	170001	206	C10H6O5
4866	D05	170578	245	C12H7NO3S
4866	E05	170621	202	C11H10N2O2
4866	F05	174027	212	C9H8O6
4866	G05	175412	238	C9H10N4O2S
4866	H05	175415	216	C8H7F3N4
4866	A06	176765	217	C9H10Cl2N2
4866	B06	177989	237	C12H15NO4
4866	C06	179818	214	C13H8ClN
4866	D06	182400	233	C6H2Cl2N4O2
4866	E06	190336	244	C8H10BrN3O
4866	F06	193043	221	C10H9ClN4
4866	G06	196148	201	C7H11N3O2S
4866	H06	197049	221	C6H7NO2S3
4866	A07	202883	203	C12H13NO2
4866	B07	204920	226	C13H10N2O2
4866	C07	204976	212	C13H12N2O
4866	D07	205843	244	C14H16N2O2
4866	E07	210816	243	C12H9N3O3
4866	F07	215276	206	C12H14O3

4866	G07	220030	217	C14H19NO
4866	H07	227383	211	C8H9N3O2S
4866	A08	234945	223	C12H9N5
4866	B08	236246	233	C11H11N3O3
4866	C08	240502	206	C8H12ClNO3
4866	D08	255025	226	C10H14N2O2S
4866	E08	261037	201	C7H11N3O4
4866	F08	261610	218	C13H18N2O
4866	G08	274905	221	C14H11N3
4866	H08	277806	230	C12H10N2O3
4866	A09	279895	236	C13H16O4
4866	B09	282187	248	C12H9FN2O3
4866	C09	284234	245	C13H11NO4
4866	D09	288519	240	C12H8N4O2
4866	E09	289090	246	C13H12ClN3
4866	F09	289365	250	C12H8ClNO3
4866	G09	290307	210	C9H10N2O4
4866	H09	292826	250	C14H16F2N2
4866	A10	293334	210	C10H14N2O3
4866	B10	293780	209	C14H11NO
4866	C10	294154	212	C8H6ClN3O2
4866	D10	294623	242	C13H14N4O
4866	E10	295404	230	C12H10N2OS
4866	F10	298197	221	C9H7N3O2S
4866	G10	298793	233	C14H19NO2
4866	H10	301168	236	C10H6ClN3S
4866	A11	309971	242	C9H10N2O2S2
4866	B11	311074	226	C6H6N6O2S
4866	C11	311727	240	C7H4N4O2S2
4866	D11	319034	229	C11H23N3S
4866	E11	321506	245	C9H6Cl2N2S
4866	F11	327693	248	C5H8N6O2S2
4866	G11	329676	214	C7H10N4O4
4866	H11	330497	206	C12H18N2O
4867	A02	332473	220	C9H6ClN5
4867	B02	335048	239	C13H9N3O2
4867	C02	338106	240	C11H8N6O
4867	D02	341074	241	C6H7N7O2S
4867	E02	343343	224	C10H6ClNOS
4867	F02	343344	204	C10H8N2OS
4867	G02	343783	238	C9H10N4S2

4867	H02	353451	243	C7H7ClN6O2
4867	A03	357683	223	C10H9NO5
4867	B03	366807	223	C9H9N3S2
4867	C03	367428	220	C7H9FN2O5
4867	D03	367487	205	C9H7N3OS
4867	E03	370367	211	C9H7ClN2S
4867	F03	372134	243	C11H15ClN2O2
4867	G03	372221	230	C12H14N4O
4867	H03	373427	245	C8H11N3O4S
4867	A04	373535	232	C12H12N2O3
4867	B04	375392	210	C14H14N2
4867	C04	375997	241	C12H11N5O
4867	D04	382059	220	C11H12N2OS
4867	E04	403374	229	C8H9ClN4S
4867	F04	503425	202	C7H4ClNS2
4867	G04	509563	243	C13H13N3S
4867	H04	515893	238	C13H22N2S
4867	A05	601351	240.26	C10H16N4O3
4867	B05	605333	223	C14H9NS
4867	C05	622175	203	C9H9N5O
4867	D05	636717	250	C17H14O2
4867	E05	637290	215	C13H13NO2
4867	F05	638080	220	C10H8N2O4
4867	G05	638134	203	C11H9NO3
4867	H05	643150	222	C14H10N2O
4867	A06	645987	242	C12H10N4O2
4867	B06	646976	233	C12H11NO4
4867	C06	659107	244	C13H12N2OS
4867	D06	660300	249	C11H11N3O4
4867	E06	479	267	C15H13N3O2
4867	F06	1847	262	C12H10N2O3S
4867	G06	3076	265.32	C15H15N5
4867	H06	3193	270	C12H18N2O3S
4867	A07	3247	275	C8H10AsNO5
4867	B07	4429	296.29	C12H12N10
4867	C07	5426	258.23	C14H10O5
4867	D07	5564	275	C16H21NO3
4867	E07	6137	263	C8H7BrO5
4867	F07	6145	286	C20H14O2
4867	G07	6731	254	C17H18O2
4867	H07	6821	289	C19H15NO2

4867	A08	6844	292.29	C18H12O4
4867	B08	7218	293	C17H15N3O2
4867	C08	7420	298	C13H16BrNO2
4867	D08	7572	278	C13H14N2O3S
4867	E08	7745	268	C13H16O6
4867	F08	7962	284	C18H20O3
4867	G08	9782	282	C19H22O2
4867	H08	10173	278	C15H18O5
4867	A09	10211	287	C19H13NO2
4867	B09	10768	255	C12H17NO5
4867	C09	11296	286	C14H14N4O3
4867	D09	131388	209	C9H15N5O
4867	E09	148170	227	C8H7ClN4S
4867	F09	154587	219	C11H17N5
4867	G09	159632	236	C12H12O5
4867	H09	166547	229	C8H6Cl2N4
4867	A10	294161	238	C14H14N4
4867	B10	329284	212	C8H8N2O3S
4867	C10	343230	226	C11H22N4O
4867	D10	7867	294	C12H9Cl2N5
4867	E10	8816	252	C11H16N4O3
4867	F10	11023	268	C16H16N2O2
4867	G10	11275	266	C12H18N4O3
4867	H10	11643	288	C14H14ClN5
4867	A11	306752	238	C9H8ClN5O
4867	B11	10865	260	C12H9AsO2
4867	C11	379639	245	C10H5ClN6
4867	D11	402843	244	C8H9AsO4
4867	E11	403379	203.2	C9H9N5O
4867	F11	647136	238	C15H14N2O
4867	G11	11276	266	C12H18N4O3
4867	H11	11624	277	C12H9ClN4S
4868	A02	11664	295	C17H21N5
4868	B02	11912	274	C19H18N2
4868	C02	12028	286	C17H22N2O2
4868	D02	12488	271	C15H11ClN2O
4868	E02	12633	275	C8H10AsNO5
4868	F02	12644	289	C9H12AsNO5
4868	G02	12646	267	C10H10AsNO3
4868	H02	13151	259	C15H11ClO2
4868	A03	13248	290	C16H20ClN3

4868	B03	13345	254	C10H10N2O4S
4868	C03	13434	269	C12H15NO6
4868	D03	13579	268	C12H10ClNO2S
4868	E03	13658	264	C14H16O5
4868	F03	13785	267	C10H10AsNO3
4868	G03	13800	254	C10H10N2O4S
4868	H03	14380	266	C12H18N4O3
4868	A04	14396	284	C15H16N4O2
4868	B04	14398	299	C15H17N5O2
4868	C04	15358	293	C12H11N3O4S
4868	D04	15359	264	C12H12N2O3S
4868	E04	15784	266	C17H12ClN
4868	F04	17148	286	C6H8ClN3O4S2
4868	G04	17362	266	C17H14O3
4868	H04	17507	253	C13H19NO4
4868	A05	19125	276	C12H10ClN5O
4868	B05	19136	273.72	C13H12ClN5
4868	C05	19141	288	C14H14ClN5
4868	D05	19824	275.35	C19H17NO
4868	E05	19962	274	C17H22O3
4868	F05	21333	260	C15H20N2O2
4868	G05	21603	290	C20H34O
4868	H05	21683	292	C16H16N6
4868	A06	21709	279	C12H17N5O3
4868	B06	21710	277	C13H19N5O2
4868	C06	22801	280	C17H16N2O2
4868	D06	22806	262	C15H22N2O2
4868	E06	22881	266	C18H18O2
4868	F06	25678	277.32	C18H15NO2
4868	G06	25740	298.32	C11H14N4O4S
4868	H06	26692	296.33	C17H16N2O3
4868	A07	28377	269	C10H15N5O2S
4868	B07	29073	276	C16H24N2O2
4868	C07	30813	272	C17H12N4
4868	D07	30930	267.28	C16H13NO3
4868	E07	31069	297	C18H19NO3
4868	F07	31698	265	C17H15NO2
4868	G07	31703	295	C18H17NO3
4868	H07	33010	253	C16H15NS
4868	A08	34875	272	C15H12O5
4868	B08	34879	267	C16H13NO3

4868	C08	34910	291	C19H17NO2
4868	D08	36586	270.24	C15H10O5
4868	E08	36753	274	C14H14N2O4
4868	F08	36815	264	C12H16N4O3
4868	G08	37612	269.34	C17H19NO2
4868	H08	37955	261	C16H23NO2
4868	A09	38352	295	C20H25NO
4868	B09	39047	256	C14H12N2O3
4868	C09	39938	281	C15H7NO3S
4868	D09	40275	263	C17H13NO2
4868	E09	40306	298	C17H12ClNO2
4868	F09	41066	286	C14H10N2O5
4868	G09	41376	278	C12H11AsO3
4868	H09	41378	277	C12H12AsNO2
4868	A10	41649	260	C16H18ClN
4868	B10	41805	300	C14H12N4O2S
4868	C10	42096	294	C12H11AsO4
4868	D10	42135	280	C10H10BrN5
4868	E10	43308	288	C18H12N2O2
4868	F10	43344	300	C16H13O4P
4868	G10	44556	287	C11H8Cl2N2OS
4868	H10	45086	261	C12H11N3O4
4868	A11	45536	266	C16H11FN2O
4868	B11	45745	261	C12H11N3O4
4868	C11	46212	298	C18H22N2O2
4868	D11	47522	255	C10H8Cl2N4
4868	E11	49643	292	C16H20O5
4868	F11	49847	274	C8H7AsO2S2
4868	G11	50199	260	C8H10BrN3S
4868	H11	51349	283	C16H13NO4
4869	A02	51351	284	C17H16O4
4869	B02	52241	284	C21H16O
4869	C02	55172	285	C13H19NO6
4869	D02	55453	272	C13H12N4OS
4869	E02	55845	285	C16H13ClN2O
4869	F02	55862	298	C21H14O2
4869	G02	58347	281	C11H9ClN4O3
4869	H02	58724	256	C14H12N2O3
4869	A03	59430	284	C11H10ClN3O2S
4869	B03	59782	252	C15H16N4
4869	C03	59984	265	C12H15N3O4

4869	D03	60303	278.69	C13H11ClN2O3
4869	E03	61929	294	C7HCl5O2
4869	F03	62901	259	C8H10AsNO4
4869	G03	63161	264	C16H16N4
4869	H03	66020	259.69	C13H10ClN3O
4869	A04	68841	267	C16H13NO3
4869	B04	68971	273	C14H11NO5
4869	C04	70307	267	C12H17N3O4
4869	D04	72947	282	C11H10N2O7
4869	E04	73295	252	C10H12N4O4
4869	F04	73753	289	C10H6Cl2N2O2S
4869	G04	76549	261	C14H13ClN2O
4869	H04	76747	272	C19H16N2
4869	A05	78623	292	C13H12N2O6
4869	B05	78697	264	C14H8N4O2
4869	C05	82269	272	C13H12N4OS
4869	D05	87008	293.43	C15H23N3OS
4869	E05	87690	271	C12H9N5O3
4869	F05	88324	251	C6H5Br2N
4869	G05	88349	254	C5HF7N2O2
4869	H05	88402	292	C13H13N2O2PS
4869	A06	88795	253	C16H15NO2
4869	B06	89349	284	C13H11Cl2NS
4869	C06	89429	296	C14H18ClN3S
4869	D06	89759	258	C15H14O4
4869	E06	92849	278	C13H14N2O3S
4869	F06	93427	286.33	C19H14N2O
4869	G06	93945	262	C12H10N2O5
4869	H06	95909	266	C15H10N2O3
4869	A07	97865	277	C11H10F3NO2S
4869	B07	98026	300	C17H16O5
4869	C07	98049	262	C9H12ClN3O4
4869	D07	98938	280	C18H16O3
4869	E07	100058	287	C17H21NO3
4869	F07	100942	274	C10H14N2O3S2
4869	G07	101298	269	C16H19N3O
4869	H07	101345	265	C11H19N7O
4869	A08	101679	273	C14H15N3O3
4869	B08	101758	259	C8H12F3NO5
4869	C08	102554	263	C12H13N3O2S
4869	D08	103189	299	C13H12F3N3O2

4869	E08	105432	284	C12H16N2O4S
4869	F08	108783	272	C13H8N2O3S
4869	G08	109747	263	C17H13NO2
4869	H08	111847	263	C17H13NO2
4869	A09	17055	280	C17H12O4
4869	B09	20618	258	C13H14N4S
4869	C09	20619	258	C13H14N4S
4869	D09	23715	265	C12H19N5O2
4869	E09	32892	278	C14H12ClNO3
4869	F09	40749	290	C16H14N6
4869	G09	43409	266	C11H14N4O2S
4869	H09	50680	293	C18H15NO3
4869	A10	54645	283	C16H13NO4
4869	B10	54860	289	C11H6Cl2OS2
4869	C10	79887	298	C14H16ClNO4
4869	D10	107022	278.17	C12H6O8
4869	E10	33173	269	C15H9ClN2O
4869	F10	38007	256	C15H10ClNO
4869	G10	60037	286	C14H18N6O
4869	H10	66122	284	C20H16N2
4869	A11	14311	263	C12H17N5O2
4869	B11	21970	268	C21H16
4869	C11	33182	276	C11H8N4OS2
4869	D11	45815	264	C13H20N4O2
4869	E11	53934	295	C13H17N3O5
4869	F11	76478	287	C17H13N5
4869	G11	92937	261	C17H11NO2
4869	H11	110899	254	C11H10O5S
4870	A02	112541	292	C18H16N2O2
4870	B02	112547	298	C16H14N2O2S
4870	C02	112965	271	C16H14FNO2
4870	D02	116640	295.34	C20H13N3
4870	E02	116644	270	C19H14N2
4870	F02	117197	299	C14H13N5O3
4870	G02	117446	261	C11H19NO4S
4870	H02	117908	286	C15H14N2S2
4870	A03	118628	288	C16H16O5
4870	B03	119805	295	C16H13N3O3
4870	C03	120631	258	C15H14O4
4870	D03	120913	262	C14H18N2O3
4870	E03	120961	280	C14H20N2O4

4870	F03	121268	272	C16H20N2O2
4870	G03	122253	290	C18H14N2O2
4870	H03	125344	281	C15H17ClO3
4870	A04	125605	257	C14H15N3O2
4870	B04	126224	265	C12H11NO6
4870	C04	127886	278.31	C14H18N2O4
4870	D04	129220	285	C9H11N3O4.C2H4O2
4870	E04	129929	266	C16H14N2O2
4870	F04	130801	280.33	C17H16N2O2
4870	G04	130847	289	C17H11N3O2
4870	H04	133002	259	C9H10NO6P
4870	A05	133351	290	C12H10N4O5
4870	B05	134058	251	C10H9N3OS2
4870	C05	134199	294	C11H10N4O4S
4870	D05	134674	257	C6HCl4N3
4870	E05	135412	288	C20H20N2
4870	F05	135894	269	C10H12Cl3NO
4870	G05	137399	273	C19H15NO
4870	H05	137577	275	C18H17N3
4870	A06	138389	253	C12H13ClN2O2
4870	B06	138398	289	C16H23N3O2
4870	C06	139021	255	C13H9N3OS
4870	D06	140873	277.71	C13H12ClN3O2
4870	E06	140899	268	C15H12N2O3
4870	F06	141538	296	C16H24O5
4870	G06	142269	273	C14H15N3OS
4870	H06	144694	274	C18H14N2O
4870	A07	147866	282	C17H22N4
4870	B07	148832	264	C15H20O4
4870	C07	151262	285	C14H9BrN2
4870	D07	153172	272	C14H12N2O4
4870	E07	153792	269	C15H15N3O2
4870	F07	154127	281	C10H12Cl3N3
4870	G07	156957	263	C13H13NO5
4870	H07	157522	275	C14H15ClN4
4870	A08	158549	260	C16H12N4
4870	B08	159686	257	C14H11NO4
4870	C08	162188	275	C9H7F6NO2
4870	D08	163802	258	C13H10N2O4
4870	E08	164464	275	C14H11ClN2O2
4870	F08	165599	287	C10H13N3O3S2

4870	G08	165701	295.4	C17H17N3S
4870	H08	166596	268	C13H8N4OS
4870	A09	166634	267	C7H6Cl3N5
4870	B09	166846	266	C12H18N4O3
4870	C09	168221	286	C15H14N2O2S
4870	D09	168225	278	C15H14N6
4870	E09	170637	256	C14H16N4O
4870	F09	170955	291	C20H21NO
4870	G09	174084	295	C12H11BrN2O2
4870	H09	175743	268.32	C14H16N6
4870	A10	176367	257	C13H11N3OS
4870	B10	177866	259.1	C13H7BrO
4870	C10	178873	268	C13H16O6
4870	D10	179822	260	C14H12O5
4870	E10	180964	274	C16H18O4
4870	F10	193528	272	C11H16N2O4S
4870	G10	195327	271	C13H13N5S
4870	H10	197046	252	C9H8N4O3S
4870	A11	201634	274	C14H14N2O2S
4870	B11	203837	267	C15H13N3O2
4870	C11	205909	290	C14H14N2O3S
4870	D11	205913	262	C12H10N2O3S
4870	E11	207895	279.25	C11H13N5O4
4870	F11	211336	273	C11H13ClN2O4
4870	G11	213708	253	C11H9F2N3O2
4870	H11	214029	265	C17H12FNO
4871	A02	215275	296	C17H16N2OS
4871	B02	215585	266	C12H12ClN3O2
4871	C02	216183	289	C14H12FN3O3
4871	D02	216618	265	C13H19N3OS
4871	E02	236254	266	C16H14N2O2
4871	F02	238929	273	C10H6Cl2N2OS
4871	G02	240029	280	C13H16N2O5
4871	H02	241621	279	C8H11BrN2O2S
4871	A03	241998	294	C14H18N2O5
4871	B03	242557	282	C18H22N2O
4871	C03	265372	264	C12H20N6O
4871	D03	283856	262	C14H18N2OS
4871	E03	285669	254	C11H14N2O3S
4871	F03	288024	297	C11H11N3O3S2
4871	G03	289748	262	C12H14N4OS

4871	H03	294150	291	C13H17N5O3
4871	A04	294625	287	C13H13N5O3
4871	B04	294747	271	C11H17N3OS2
4871	C04	296934	297	C12H15N3O6
4871	D04	299514	288	C13H12N4O2S
4871	E04	299967	287	C14H13N3O2S
4871	F04	299968	270	C10H14N4O3S
4871	G04	301167	272	C10H7Cl2N3S
4871	H04	302584	278	C10H6N4O4S
4871	A05	302867	288	C14H16N4O3
4871	B05	303304	258	C18H14N2
4871	C05	305329	262	C15H10N4O
4871	D05	305743	258	C13H8ClN3O
4871	E05	307703	257	C14H9ClN2O
4871	F05	308814	283	C20H13NO
4871	G05	310113	278	C13H14N2O3S
4871	H05	311165	285	C16H19N3O2
4871	A06	312606	284	C15H16N4O2
4871	B06	316458	263.26	C11H13N5O3
4871	C06	318799	262	C9H12ClN3O4
4871	D06	319029	288	C17H24N2O2
4871	E06	319079	297	C14H17ClN2O3
4871	F06	319436	299	C16H17N3OS
4871	G06	319471	252	C12H16N2O2S
4871	H06	321792	254	C10H8ClN3OS
4871	A07	326644	269	C14H9ClN4
4871	B07	326921	274	C14H14N2S2
4871	C07	327444	268	C13H20N2S2
4871	D07	328130	261	C14H19N3S
4871	E07	331972	263	C13H17N3O3
4871	F07	332452	286	C11H9ClFN3OS
4871	G07	338564	277	C13H13ClN4O
4871	H07	343526	265	C12H11NO2S2
4871	A08	343557	281	C13H10Cl2N2O
4871	B08	348970	258	C13H18N6
4871	C08	351674	286	C20H18N2
4871	D08	351691	260	C16H24N2O
4871	E08	352888	279.34	C14H21N3O3
4871	F08	358311	271	C14H13N3O3
4871	G08	361570	283	C10H9N3O5S
4871	H08	364889	299	C17H17NO4

4871	A09	366086	269	C9H7N3OS3
4871	B09	366802	298	C17H22N4O
4871	C09	367474	281	C17H19N3O
4871	D09	367480	298	C16H12ClN3O
4871	E09	369066	269	C12H7N5O3
4871	F09	369070	299	C14H9N3O3S
4871	G09	122385	270	C10H10N2O3S2
4871	H09	164880	291	C19H17NO2
4871	A10	228155	290.25	C11H6N4O4S
4871	B10	294153	288	C11H8N6O4
4871	C10	362093	275	C14H11ClN2O2
4871	D10	363801	253	C15H15N3O
4871	E10	367416	282	C14H10N4OS
4871	F10	217306	290	C16H18O5
4871	G10	276736	281	C17H15NO3
4871	H10	283845	273.29	C18H11NO2
4871	A11	116508	276	C16H24N2O2
4871	B11	149312	266	C16H14N2O2
4871	C11	204939	295	C18H17NO3
4871	D11	252172	278	C19H18O2
4871	E11	269904	299	C14H16Cl2N2O
4871	F11	280492	298	C13H18N2O2S2
4871	G11	330796	283	C16H13NO4
4871	H11	331977	254	C15H14N2O2
4872	A02	370383	264	C11H12N4O4
4872	B02	372769	290	C15H9Cl2NO
4872	C02	373981	257.25	C14H11NO4
4872	D02	374703	298	C18H19O2P
4872	E02	374814	276	C15H20N2O3
4872	F02	375105	285	C19H15N3
4872	G02	378711	281	C14H17ClN2O2
4872	H02	379468	296	C13H11Cl2N3O
4872	A03	379536	296	C10H9ClF3N3O2
4872	B03	379651	277	C16H11N3O2
4872	C03	380279	295	C9H9N7O3S
4872	D03	400770	274	C19H14O2
4872	E03	400938	253	C13H11N5O
4872	F03	403268	265	C11H7NO3S2
4872	G03	408734	273	C11H11N7O2
4872	H03	408860	286	C14H10N2O5
4872	A04	522131	297	C13H10Cl2N2O2

4872	B04	524615	278	C16H22O4
4872	C04	525721	271	C15H13NO2S
4872	D04	636718	264	C18H16O2
4872	E04	637343	276	C18H16N2O
4872	F04	637359	258	C17H10N2O
4872	G04	637827	276	C19H16O2
4872	H04	638636	257	C7H16NO.I
4872	A05	641396	291	C18H13NO3
4872	B05	643029	280	C19H20O2
4872	C05	645330	275	C18H13NO2
4872	D05	661221	275	C18H13NO2
4872	E05	680515	265	C16H15N3O
4872	F05	689002	287	C14H9NO4S
4872	G05	3753	304.36	C15H16N2O3S
4872	H05	5053	335	C19H15N4.Cl
4872	A06	5476	334	C18H26N2O2S
4872	B06	6101	330	C20H26O4
4872	C06	7436	304.41	C16H20N2O2S
4872	D06	7578	343	C21H13NO4
4872	E06	9037	336.3	C19H12O6
4872	F06	11437	302	C17H16ClNO2
4872	G06	12262	314	C18H22N2O3
4872	H06	12544	320	C15H10ClNO3S
4872	A07	12628	321	C8H8AsNO8
4872	B07	12650	322	C12H11AsN2O4
4872	C07	13156	314.36	C15H14N4O2S
4872	D07	13176	318	C22H26N2
4872	E07	13616	327.47	C20H29N3O
4872	F07	14142	327	C16H17N5O3
4872	G07	14506	332	C17H33O4P
4872	H07	16722	302	C16H14O6
4872	A08	17128	348	C21H29FO3
4872	B08	18883	349	C22H24N2S
4872	C08	24032	310	C22H18N2
4872	D08	24951	350	C21H22N2O3
4872	E08	25435	310.29	C18H15O3P
4872	F08	25457	348.4	C24H16N2O
4872	G08	26349	302	C18H22O4
4872	H08	26980	334.33	C15H18N4O5
4872	A09	27305	324	C13H16N4O4S
4872	B09	28080	312	C19H20O4

4872	C09	30205	314.39	C21H18N2O
4872	D09	30260	349.42	C15H15N3O3S2
4872	E09	32673	334	C21H22N2O2
4872	F09	32873	305	C9H12AsNO6
4872	G09	33353	337.81	C19H16ClN3O
4872	H09	33478	328	C19H24N2O3
4872	A10	33738	339	C15H12Cl2N2O3
4872	B10	34219	313	C16H21ClO4
4872	C10	34865	322	C12H11AsN2O4
4872	D10	35582	347	C15H10N2S4
4872	E10	36693	332.44	C20H28O4
4872	F10	36758	305.82	C15H15N3S.ClH
4872	G10	36923	317	C20H31NO2
4872	H10	37168	308.29	C17H12N2O4
4872	A11	37187	312	C18H14ClNO2
4872	B11	38090	333	C14H11N3O5S
4872	C11	39984	314	C17H12ClNO3
4872	D11	40269	326	C22H18N2O
4872	E11	41098	312	C18H14ClNO2
4872	F11	43088	335	C19H13NO3S
4872	G11	43506	315	C14H20ClN2O2P
4872	H11	44584	334	C12H14N8O2S
4873	A02	44750	312	C15H8N2O6
4873	B02	45527	304.37	C14H16N4O2S
4873	C02	46213	312	C19H24N2O2
4873	D02	46492	313	C17H19N3O3
4873	E02	48388	330	C12H10N8S2
4873	F02	50648	308	C17H12N2O4
4873	G02	50651	336.78	C19H13ClN2O2
4873	H02	50690	307.35	C19H17NO3
4873	A03	51683	315.8	C17H18ClN3O
4873	B03	55152	346	C20H18N4O2
4873	C03	59814	317.34	C20H15NO3
4873	D03	60013	307	C14H8Cl2N2O2
4873	E03	60183	310	C14H18N2O4S
4873	F03	60423	321	C16H17ClN2O3
4873	G03	61642	344	C18H16N8
4873	H03	62375	310	C16H30N4O2
4873	A04	64672	344	C15H16N6O4
4873	B04	65689	327	C19H21NO4
4873	C04	68116	315	C11H21Cl2N2O2P

4873	D04	69359	302	C19H14N2O2
4873	E04	70413	327.38	C19H21NO4
4873	F04	71097	310	C18H12ClNO2
4873	G04	71866	305	C11H9BrN6
4873	H04	71881	314.35	C19H14N4O
4873	A05	76988	302.24	C15H10O7
4873	B05	79486	340	C21H28N2O2
4873	C05	79559	308	C18H16N2O3
4873	D05	81750	329.31	C15H15N5O4
4873	E05	82560	309	C10H11N7O3S
4873	F05	83497	326	C19H22N2OS
4873	G05	86467	328	C20H24O4
4873	H05	87084	331	C17H17NO4S
4873	A06	87136	312	C19H24N2O2
4873	B06	87838	346.43	C22H22N2O2
4873	C06	90749	303.38	C15H17N3O2S
4873	D06	91378	342	C20H26N2O3
4873	E06	91382	340	C21H28N2O2
4873	F06	92892	337	C13H12AsNO5
4873	G06	93033	305.25	C13H11N3O6
4873	H06	95204	306	C10H19N4O3PS
4873	A07	95916	343	C16H17N5O4
4873	B07	653004	291	C17H25NO3
4873	C07	1014	341	C22H19N3O
4873	D07	22070	306	C17H22O5
4873	E07	26112	306	C13H8Cl4
4873	F07	33570	321	C21H23NO2
4873	G07	36525	342.4	C18H22N4O3
4873	H07	43271	304	C12H10BrN5
4873	A08	53874	327	C8H11Cl2NO3.C4H9NO
4873	B08	56779	304	C15H13N3O2.ClH
4873	C08	87010	302	C16H16ClN3O
4873	D08	636734	279	C17H13NO3
4873	E08	6268	318.33	C18H14N4O2
4873	F08	11307	314	C16H18N4O3
4873	G08	26113	318	C14H8Cl4
4873	H08	35545	301	C18H23NO3
4873	A09	43998	318	C18H14N4O2
4873	B09	47680	301	C12H10Cl2N2OS
4873	C09	48443	332	C20H32N2O2
4873	D09	66695	340	C12H10BrN3O4

4873	E09	73254	347	C22H25N3O
4873	F09	80313	325.33	C20H11N3O2
4873	G09	94600	348	C20H16N2O4
4873	H09	524385	268	C11H16N4O2S
4873	A10	680516	290	C18H18N4
4873	B10	30622	333	C18H23NO5
4873	C10	19123	305	C12H9ClN6O2
4873	D10	3064	328	C16H16N4O4
4873	E10	33575	345.44	C23H23NO2
4873	F10	45545	308	C16H12N4O3
4873	G10	48617	312	C20H28N2O
4873	H10	60659	312.37	C20H16N4
4873	A11	63543	326	C21H26O3
4873	B11	64859	328	C10H6Cl4N2O2
4873	C11	70895	316	C12H15Cl2N5O
4873	D11	76015	344	C7H6Br3N
4873	E11	81493	303	C13H13N5O2S
4873	F11	81856	327	C12H8Cl2N4O3
4873	G11	83961	303	C14H11ClN4O2
4873	H11	25673	347	C17H12Cl2N2O2
4874	A02	96541	310	C21H14N2O
4874	B02	96996	339	C22H17N3O
4874	C02	99634	322	C14H14N2O5S
4874	D02	99660	339.47	C14H21N5OS2
4874	E02	99663	319	C13H13N5OS2
4874	F02	101789	311	C22H17NO
4874	G02	105348	330	C18H16ClNO3
4874	H02	105781	304	C19H16N2O2
4874	A03	105798	328	C17H16N2O5
4874	B03	105827	325.34	C12H15N5O4S
4874	C03	106464	332	C17H20N2O5
4874	D03	110332	303	C16H21N3O3
4874	E03	110562	302	C10H8BrNO3S
4874	F03	111194	347	C18H19ClN2OS
4874	G03	112203	311	C15H21NO6
4874	H03	114449	338.34	C12H14N6O4S
4874	A04	114997	326	C18H18N2O4
4874	B04	116397	329	C20H27NO3
4874	C04	120290	332	C12H8N6O2S2
4874	D04	120622	316	C16H16N2O5
4874	E04	121908	306	C16H22N2O2S

4874	F04	123389	325	C19H19NO4
4874	G04	126226	302	C15H14N2O5
4874	H04	126347	328	C13H16N2O2S3
4874	A05	129536	310	C15H18O7
4874	B05	133114	346.32	C10H14N6O6S
4874	C05	135184	308	C15H20N2O3S
4874	D05	137112	347	C21H21N3O2
4874	E05	142277	320	C15H13FN2O3S
4874	F05	143974	336	C15H25N6OP
4874	G05	146071	320	C11H5Cl3N2O3
4874	H05	146554	350.35	C14H14N4O5S
4874	A06	146769	334	C18H14N4O3
4874	B06	146770	304	C18H16N4O
4874	C06	147358	301	C17H17ClN2O
4874	D06	149054	307	C17H13N3O3
4874	E06	153391	328.38	C17H16N2O3S
4874	F06	154585	335	C19H17N3O3
4874	G06	157725	329.74	C16H12ClN3O3
4874	H06	158959	316	C17H11Cl2NO
4874	A07	159092	332.8	C16H13ClN2O2S
4874	B07	159398	347	C5H3I2NO
4874	C07	164435	338	C19H16ClN3O
4874	D07	164459	305	C15H13ClN2O3
4874	E07	165704	336	C19H16N2O4
4874	F07	169409	339	C17H25NO6
4874	G07	176736	335	C13H7Cl4NO
4874	H07	177407	333.09	C12H5Cl2F3N4
4874	A08	186067	339.44	C20H25N3O2
4874	B08	186194	324	C14H20N4O5
4874	C08	186200	340	C23H20N2O
4874	D08	190501	326	C13H10BrClN2O
4874	E08	191441	347	C21H17NO4
4874	F08	194308	306	C12H22N2O3S2
4874	G08	201659	323	C14H11ClN2O3S
4874	H08	201989	343	C19H22N2S2
4874	A09	202705	305	C13H9ClN4OS
4874	B09	205827	301	C15H15N3O2S
4874	C09	205832	350	C18H20ClNO4
4874	D09	205842	319	C15H17N3O3S
4874	E09	205912	325	C12H11N3O4S2
4874	F09	211340	303	C12H9N5O3S

4874	G09	211787	326	C18H18N2O4
4874	H09	215684	318	C15H14N2O6
4874	A10	215689	345	C16H15N3O6
4874	B10	216607	320	C15H13FN2O5
4874	C10	216621	350	C20H31NO2S
4874	D10	216623	301	C15H15N3O2S
4874	E10	228137	334	C15H14N2O5S
4874	F10	228150	303	C13H9N3O4S
4874	G10	241619	305	C14H15N3O5
4874	H10	241624	332	C17H20N2O3S
4874	A11	244387	328	C17H28O6
4874	B11	246999	331	C21H14FNS
4874	C11	270063	308	C15H14ClNO4
4874	D11	270916	337	C22H27NO2
4874	E11	281307	331	C12H10Cl3N5
4874	F11	281383	319	C16H21N3O2S
4874	G11	281623	340	C18H16N2O3S
4874	H11	281624	310	C18H18N2OS
4875	A02	282137	314	C17H22N4O2
4875	B02	283849	328	C18H20N2O4
4875	C02	288387	348	C19H16N4O3
4875	D02	293360	343	C18H17NO6
4875	E02	294750	310	C11H10N4O3S2
4875	F02	294756	333	C16H19N3OS2
4875	G02	295300	330	C20H18N4O
4875	H02	299119	312	C12H16N4O2S2
4875	A03	300540	331	C17H12Cl2N2O
4875	B03	303294	347	C15H13N3O3S2
4875	C03	303612	322	C20H22N2S
4875	D03	305780	341	C18H14N4S.1/2C2H6O
4875	E03	308848	311	C18H21N3O2
4875	F03	308849	309	C18H19N3O2
4875	G03	319012	321	C16H23N3O4
4875	H03	319424	309	C10H4F5N3OS
4875	A04	319449	341	C19H19NO3S
4875	B04	321517	313	C14H23N3OS2
4875	C04	326182	309.32	C17H15N3O3
4875	D04	326385	334	C10H5ClINO2
4875	E04	326422	306	C14H18N4O2S
4875	F04	326757	318.33	C20H14O4
4875	G04	328010	336	C16H20N2O6

4875	H04	328111	325	C13H6Cl2N2O4
4875	A05	329052	322	C15H22N4O4
4875	B05	329255	329	C16H15N3OS2
4875	C05	330770	311	C16H17N5O2
4875	D05	332670	310.35	C21H14N2O
4875	E05	333544	336	C16H24N4O2S
4875	F05	335504	337	C16H17ClN2O4
4875	G05	339589	322	C15H16ClN3OS
4875	H05	339594	339	C16H13N5O2S
4875	A06	339630	339	C15H21N3.CH4O3S
4875	B06	341956	307.3	C15H17NO6
4875	C06	343550	309	C11H9BrN4S
4875	D06	345850	332	C16H20N4O4
4875	E06	346578	339	C20H18FNO3
4875	F06	349156	325	C14H15NO8
4875	G06	352890	302.72	C11H14N4O4.ClH
4875	H06	362639	344.24	C13H8N6O6
4875	A07	366289	303	C10H6Cl3N5
4875	B07	366801	333	C17H21ClN4O
4875	C07	369986	329	C15H19N7O2
4875	D07	371765	318	C17H14N6O
4875	E07	372146	305	C18H15N3O2
4875	F07	372275	322	C19H18N2O3
4875	G07	372287	339	C17H13N3O5
4875	H07	135381	304	C17H20O3S
4875	A08	145992	301	C12H13ClN2O5
4875	B08	187675	323.14	C12H10AsNO5
4875	C08	201868	318	C19H18N4O
4875	D08	209901	331	C13H6Cl3NOS
4875	E08	217913	307	C9H8Cl2N4O2S
4875	F08	326184	316	C15H10ClN3O3
4875	G08	329249	348.76	C14H9ClN4O3S
4875	H08	337726	304	C15H16N2O3S
4875	A09	343549	301	C19H27NO2
4875	B09	211356	346	C16H18N4O5
4875	C09	222362	319	C12H16AsClO3
4875	D09	252359	342.83	C17H19ClN6
4875	E09	300289	339.35	C18H17N3O4
4875	F09	321502	346	C17H9Cl2NOS
4875	G09	324623	327	C20H13N3O2
4875	H09	98363	342	C12H10N2O4S3

4875	A10	99657	325	C13H19N5OS2
4875	B10	111118	331	C13H8Cl2S3
4875	C10	112125	312	C18H12N6
4875	D10	133075	343.36	C15H13N5O3S
4875	E10	135168	313	C21H15NO2
4875	F10	142446	349.82	C19H16ClN5
4875	G10	150114	303	C21H21NO
4875	H10	156563	350.33	C19H14N2O5
4875	A11	197008	336	C18H28N2O4
4875	B11	245091	324	C22H28O2
4875	C11	278323	326	C10H18N2O6S2
4875	D11	326375	338	C23H18N2O
4875	E11	329250	349	C14H9ClN4O3S
4875	F11	331968	301	C16H10Cl2N2
4875	G11	338578	318	C18H14N4O2
4875	H11	339316	303	C15H21N5S
4876	A02	372499	308	C18H16N2O3
4876	B02	375981	337.33	C18H15N3O4
4876	C02	375982	344	C19H28N4O2
4876	D02	378717	333	C17H19NO2S2
4876	E02	379538	304	C13H10ClN5S
4876	F02	379697	343	C16H11ClN4OS
4876	G02	401077	334.33	C19H14N2O4
4876	H02	403447	304	C19H20N4
4876	A03	623109	307	C9H13BrClN5
4876	B03	623638	350	C21H22N2O3
4876	C03	631160	317	C12H11N7O2S
4876	D03	632536	323.48	C21H29N3
4876	E03	637153	327	C14H15BrO4
4876	F03	637317	344	C13H15BrNO3P
4876	G03	637325	310	C13H15N2O5P
4876	H03	651084	347	C16H17N3O6
4876	A04	660151	304	C15H17N2O3P
4876	B04	3323	375	C23H25N3O2
4876	C04	5157	395.41	C22H21NO6
4876	D04	5836	362	C17H18N2O3S2
4876	E04	7419	393	C13H15Cl5N2O
4876	F04	11668	385.25	C18H14Cl2N6
4876	G04	12666	364	C14H13AsN2O5
4876	H04	13487	379	C27H26N2
4876	A05	14974	396	C20H28O8

4876	B05	16437	368	C24H16O4
4876	C05	16736	362.34	C18H18O8
4876	D05	17355	357	C20H23NO5
4876	E05	19061	385.25	C18H14Cl2N6
4876	F05	20192	386	C21H39NO5
4876	G05	24113	369	C21H21ClN2S
4876	H05	29200	357	C17H13F2N5O2
4876	A06	31748	358	C7H8AsIO4
4876	B06	34871	364	C14H13AsN2O5
4876	C06	36317	384	C20H24N4O4
4876	D06	36818	374.48	C19H30N6O2
4876	E06	46075	370	C20H20ClN3O2
4876	F06	46385	390.42	C16H18N6O4S
4876	G06	49852	384	C12H9AsN2O8
4876	H06	50650	363	C21H17NO5
4876	A07	50654	382.42	C24H18N2O3
4876	B07	50688	358	C19H16ClNO4
4876	C07	57624	384	C22H28N2O4
4876	D07	65537	356.4	C17H16N4O3S
4876	E07	70799	377	C22H36N2O3
4876	F07	73053	381	C17H17NO3.H2O4S
4876	G07	76350	398	C21H22N2O6
4876	H07	78846	359	C20H29N3O3
4876	A08	80137	386.49	C22H30N2O4
4876	B08	81463	400	C22H28N2O5
4876	C08	81915	378	C10H10BrN5.H2O4S
4876	D08	84100	392.46	C26H20N2O2
4876	E08	89602	377	C20H25ClN2O3
4876	F08	91340	381	C19H22Cl2N2O2
4876	G08	91355	361	C20H25ClN2O2
4876	H08	91356	361	C20H25ClN2O2
4876	A09	91357	361	C20H25ClN2O2
4876	B09	91368	377	C20H25ClN2O3
4876	C09	91396	397	C23H25ClN2O2
4876	D09	91397	396.92	C23H25ClN2O2
4876	E09	97920	392.5	C26H24N4
4876	F09	99867	363	C20H25N7
4876	G09	99925	383	C21H21NO6
4876	H09	100708	385	C23H22F3NO
4876	A10	103331	399	C17H20Cl2N4OS
4876	B10	106231	360	C23H24N2O2

4876	C10	107679	379.88	C13H18ClN3O4S2
4876	D10	110300	358	C16H14N4O2S2
4876	E10	111210	396.92	C23H25ClN2O2
4876	F10	114414	357	C16H15N5O3S
4876	G10	117268	378.84	C15H15ClN6O2S
4876	H10	117987	360	C21H12O4S
4876	A11	118818	369	C23H19N3O2
4876	B11	124818	354	C12H8BrN3OS2
4876	C11	126837	391	C19H21NO6S
4876	D11	131467	386	C23H18N2O4
4876	E11	131616	354	C21H26N2O3
4876	F11	134137	371.37	C17H13N3O5S
4876	G11	139168	390	C22H30O6
4876	H11	151721	354	C18H26O7
4877	A02	151888	375	C25H30N2O
4877	B02	154389	396	C18H10Cl4N2
4877	C02	156565	359.38	C22H17NO4
4877	D02	159566	351	C16H15BrO4
4877	E02	163639	399	C12H12Cl2N2O7S
4877	F02	163823	356	C14H9ClF3N5O
4877	G02	164991	367	C16H12Cl2N2O2S
4877	H02	166637	384	C13H14Cl2F3N5O
4877	A03	177862	351	C21H21NO4
4877	B03	191454	363	C21H17NO5
4877	C03	201863	370.28	C21H17Cl2NO
4877	D03	204262	396	C17H15Cl2N3O2S
4877	E03	204665	376	C20H22ClNO4
4877	F03	215721	386	C14H10BrClN2O2S
4877	G03	216606	354	C15H13BrFNO3
4877	H03	217697	359	C25H30N2
4877	A04	250429	364	C20H28O6
4877	B04	263220	387.86	C14H14ClN3O4S2
4877	C04	270071	391	C16H17N5O3S2
4877	D04	271923	359.44	C14H21N3O2.CH4O3S
4877	E04	275266	374	C19H22N2O6
4877	F04	275971	365	C16H13ClN2O2S2
4877	G04	289359	352	C19H16N2O5
4877	H04	292140	373	C17H10Cl2N4O2
4877	A05	292923	370	C18H12BrNO3
4877	B05	293962	366	C17H27N5O2S
4877	C05	298892	373	C20H15N5O3

4877	D05	309401	377	C17H16N6.2ClH
4877	E05	310354	372	C21H29N3OS
4877	F05	317605	368	C21H22ClN3O
4877	G05	319435	367	C19H17N3O3S
4877	H05	319994	362	C19H18N6O2
4877	A06	320218	388	C22H20N4O3
4877	B06	325014	383	C14H20Cl2N2O6
4877	C06	329065	369	C20H23N3O2S
4877	D06	338519	355	C20H16F3N3
4877	E06	339161	395	C20H15ClN4O3
4877	F06	347463	357	C18H15NO5S
4877	G06	630602	347	C22H21NO3
4877	H06	634396	347	C17H15ClN2O2S
4877	A07	37219	358	C24H22O3
4877	B07	56287	365	C9H7BrClN5O2S
4877	C07	102314	358	C22H30O4
4877	D07	116702	362.43	C25H18N2O
4877	E07	116709	353	C24H19NO2
4877	F07	120289	390.43	C16H14N4O4S2
4877	G07	372767	345	C15H9BrN2O3
4877	H07	407628	302	C20H14O3
4877	A08	32984	369	C21H23NO5
4877	B08	37627	392.41	C26H16O4
4877	C08	45572	386	C18H18N4O4S
4877	D08	63680	376.46	C21H24N6O
4877	E08	117028	398.3	C20H20BrN3O
4877	F08	216633	391	C18H16Cl2N4O2
4877	G08	275428	361.83	C18H19N3O3.ClH
4877	H08	295486	359	C22H15ClN2O
4877	A09	345845	364.45	C24H20N4
4877	B09	622608	322.24	C9H11N3OS2.BrH
4877	C09	622691	317	C10H11N3OS4
4877	D09	637578	325	C20H15N5
4877	E09	680495	312	C15H10BrN3
4877	F09	4292	380.44	C19H12N2O3S2
4877	G09	9032	364	C25H20N2O
4877	H09	11881	390	C23H23N3OS
4877	A10	60785	363.54	C25H33NO
4877	B10	62685	353	C10H6Cl2N2O6S
4877	C10	84126	385	C11H12AsN5O4S
4877	D10	88600	377	C19H21ClN2O2S

4877	E10	88916	369.56	C23H31NOS
4877	F10	125095	375	C18H14FNO5S
4877	G10	142335	398.85	C23H15ClN4O
4877	H10	163443	394.47	C26H22N2O2
4877	A11	178249	354	C19H14O7
4877	B11	214009	362	C21H19FN4O
4877	C11	280594	400.29	C13H17N6O7P
4877	D11	319709	352	C21H25N3S
4877	E11	321491	383	C20H15ClN2O2S
4877	F11	327702	353.44	C22H15N3S
4877	G11	338042	392.46	C26H20N2O2
4877	H11	54709	369.55	C24H35NO2
4878	A02	350187	388	C16H25N2O5PS
4878	B02	354261	355	C19H21N3O4
4878	C02	359472	361.4	C20H19N5O2
4878	D02	367306	381	C14H6Cl2F4N2O2
4878	E02	367469	362	C14H16ClNO2S3
4878	F02	379388	359	C18H18FN3O2S
4878	G02	379555	357.81	C17H12ClN3O2S
4878	H02	382035	378.43	C20H22N6O2
4878	A03	601359	392.25	C21H14BrNO2
4878	B03	603071	363.37	C20H17N3O4
4878	C03	607097	357.41	C19H23N3O4
4878	D03	614826	361.4	C22H19NO4
4878	E03	622689	353	C10H10ClN3S2.BrH
4878	F03	661122	370	C22H26O5
4878	G03	665497	368	C20H16O7
4878	H03	670283	356.46	C25H24O2
4878	A04	672865	367	C17H16F2NO4P
4878	B04	5856	448.56	C23H20N4O2S2
4878	C04	12865	405	C20H24N2O2.BrH
4878	D04	13294	430	C22H15Cl3N2O
4878	E04	13791	447	C12H11As2NO8
4878	F04	30625	411	C21H33NO7
4878	G04	41148	414	C22H22O8
4878	H04	42199	404	C21H25NO.CH4O3S
4878	A05	53275	434.45	C23H22N4O5
4878	B05	57608	401	C20H36N2O6
4878	C05	58904	431.45	C24H21N3O5
4878	D05	65238	408	C20H31N.C7H6O2
4878	E05	70933	415	C18H19ClN2.C4H4O4

4878	F05	85433	424	C17H12Br2O3
4878	G05	89201	440.41	C23H31Cl2NO3
4878	H05	89821	407	C20H27ClN4O3
4878	A06	105584	422	C21H26O9
4878	B06	107582	430	C15H7BrF3N3O4
4878	C06	107677	409	C20H24N2.C4H4O4
4878	D06	109128	416	C25H37NO4
4878	E06	123418	441	C22H23N3O7
4878	F06	123527	412	C26H21NO2S
4878	G06	127133	434.45	C27H18N2O4
4878	H06	128606	435.43	C27H17NO5
4878	A07	136513	410.38	C22H18O8
4878	B07	143241	411	C23H25NO6
4878	C07	164676	404	C20H25N3O4S
4878	D07	172255	424	C17H23Cl2NO.CH4O3S
4878	E07	201631	436.44	C21H16N4O5S
4878	F07	204232	406.35	C20H14N4O6
4878	G07	215718	401.28	C18H13BrN2O2S
4878	H07	234348	421	C19H21ClN4O5
4878	A08	280058	430	C22H22O9
4878	B08	290311	421	C27H36N2O2
4878	C08	295358	408	C16H18F6N6
4878	D08	305798	404.33	C19H18F6N2O
4878	E08	309874	446.99	C26H23ClN2OS
4878	F08	309892	407.87	C21H14ClN3O2S
4878	G08	321496	410.99	C25H31ClN2O
4878	H08	328087	414	C21H17Cl2N3O2
4878	A09	328403	427	C23H25NO7
4878	B09	335506	417.93	C18H21ClN2.C4H7NO3
4878	C09	337832	430	C14H20N6O3.C2H6O3S
4878	D09	373600	401	C17H27N3O6S
4878	E09	376254	447	C21H21NO8S
4878	F09	378719	404.25	C20H15Cl2NO4
4878	G09	379099	402	C20H13Cl2NO4
4878	H09	8675	492	C31H42N3.Cl
4878	A10	15910	451	C10H6Cl6O5S
4878	B10	19803	464.38	C21H20O12
4878	C10	36508	471	C26H30O8
4878	D10	37553	476.58	C30H28N4O2
4878	E10	37641	496.57	C29H33FO6
4878	F10	41400	493	C24H24AsN3O4

4878	G10	55691	493.23	C18H11Br2N3S2
4878	H10	60339	486.96	C26H23ClN6O2
4878	A11	64876	495	C25H32Cl2N2O4
4878	B11	70931	451	C29H38O4
4878	C11	73735	484.51	C28H24N2O6
4878	D11	80997	472.63	C30H36N2O3
4878	E11	103520	462	C17H14Br2N6
4878	F11	107522	499	C21H20Cl2N2O8
4878	G11	116339	458.55	C26H34O7
4878	H11	146771	476.49	C27H20N6O3
4879	A02	166375	464	C15H25ClN2O.C6H13NO3S
4879	B02	168184	467	C12H18N6.2C2H6O3S
4879	C02	196515	476	C20H34O5.C4H11NO3
4879	D02	211490	472.49	C28H24O7
4879	E02	281816	472.62	C20H24N2S2.C4H4O4
4879	F02	292253	464	C21H21NO5.CH4O3S
4879	G02	317003	456	C18H20N2O2S.HI
4879	H02	319990	474.55	C23H18N6O2S2
4879	A03	322661	475.3	C16H15Cl2F3N2O.CH4O3S
4879	B03	335979	452	C25H29N3O5
4879	C03	342459	469	C25H32N4O5
4879	D03	343256	478	C29H35NO5
4879	E03	371178	500	C29H33N5O3
4879	F03	379696	499	C19H16Cl2N4O4S2
4879	G03	31762	529	C19H17I2NO
4879	H03	45384	520.5	C26H24N4O8
4879	A04	80731	507.37	C26H20Cl2N4O3
4879	B04	80735	528.48	C26H20N6O7
4879	C04	91529	516.46	C25H24O12
4879	D04	133071	512.02	C20H24ClN5O2.C2H6O3S
4879	E04	139105	539	C21H25ClN6O2.C2H6O3S
4879	F04	202386	521.49	C27H19N7O5
4879	G04	345647	546.53	C30H26O10
4879	H04	354844	508.52	C28H28O9
4879	A05	654260	549.81	C22H21BrN6O4.ClH
4879	B05	679525	533	C27H20N2O8S
4879	C05	727038	542	C34H43N3O3
4879	D05	143491	579	C27H30N2O10.ClH
4879	E05	177365	566.46	C23H23N7O4S.2ClH
4879	F05	268251	575.75	C29H49N7O5
4879	G05	330500	560.69	C30H44N2O8

4879	H05	122819	656.66	C32H32O13S
4879	A06	227186	697.14	C35H37ClN2O11
4879	B06	19990	770	C40H51NO14
4879	C06	1614	446.63	C27H42O5
4879	D06	13051	407	C24H23ClN2O2
4879	E06	59620	413	C26H36O4
4879	F06	107701	416	C24H21N3O2S
4879	G06	156516	425	C26H16O2S2
4879	H06	11667	474.16	C18H14Br2N6
4879	A07	159242	463	C28H30O6
4879	B07	277184	454	C18H20N4O4.H2O4S
4879	C07	310325	502	C25H22Cl2N2OS2
4879	D07	30663	405	C26H32N2O2
4879	E07	158413	429.91	C21H13ClFNO2S2
4879	F07	222365	435	C16H17AsCl2O5
4879	G07	341196	410.47	C22H26N4O4
4879	H07	19970	499	C27H34N2O7
4879	A08	3391	554	C23H26N2O4.C7H13NO3
4879	B08	380802	363	C19H17N5O3
4879	C08	645033	372	C18H15Cl2N5
4879	D08	121868	422.53	C28H26N2O2
4879	E08	163910	441.31	C18H11N5O9
4879	F08	166259	416	C19H17ClN2OS.1/2C4H6O4
4879	G08	168027	424	C21H28O9
4879	H08	203912	410	C14H8Cl4N2O4
4879	A09	311153	433.55	C24H28N3O.C2H3O2
4879	B09	638432	407	C24H22N2O2.ClH
4879	C09	5907	463	C22H18N2O2.2C2H4O2
4879	D09	67436	486.96	C26H23ClN6O2
4879	E09	96021	455.68	C29H45NO3
4879	F09	146557	481	C24H18BrFN2OS
4879	G09	308835	484.59	C30H32N2O4
4879	H09	260594	504.55	C29H24N6O3
