# Supplementary material for: Cryptosporidium parvum Pyruvate Kinase Inhibitors With in vivo Anti-cryptosporidial Efficacy
Source: Front Microbiol. 2022 Jan 3;12:800293. doi: 10.3389/fmicb.2021.800293 (PMC8761912; doi:10.3389/fmicb.2021.800293)
Supplement: Supplementary file 4 [file Table_2.PDF]

Supplementary Table S2. Physicochemical properties of identified CpPyK inhibitors (Adapted from: *https://pubchem.ncbi.nlm.nih.gov*).

NSC number	IUPAC name	Molecular formula	Molecular weight	cLog(*p*)	Number of Hydrogen bond donors	Number of Hydrogen bond acceptors	Number of rotatable bonds	Polar surface area (Å²)
234945	2-(3-pyridin-2-yl-1*H*-1,2,4-triazol-5-yl)pyridine	$C_{12}H_9N_5$	223.23	1.2	1	4	2	67.4
252172	(2*E*)-2-[(4-ethoxyphenyl)methylidene]-3,4-dihydronaphthalen-1-one	$C_{19}H_{18}O_2$	278.3	4.4	0	2	3	26.3
636718	(2*E*)-2-[(3-methoxyphenyl)methylidene]-3,4-dihydronaphthalen-1-one	$C_{18}H_{16}O_2$	264.3	4.1	0	2	2	26.3
303244	4,6,7-trimethyl-1,4-dihydroquinazoline-8-carbonitrile	$C_{12}H_{13}N_3$	199.25	1.6	1	2	0	48.2
638080	(*E*)-2-cyano-3-(3,4,5-trihydroxyphenyl)prop-2-enamide	$C_{10}H_8N_2O_4$	220.18	0.1	4	5	2	128
11437	(*E*)-1-(5-chloro-2-hydroxyphenyl)-3-[4-(dimethylamino)phenyl]prop-2-en-1-one	$C_{17}H_{16}ClNO_2$	301.8	4.6	1	3	4	40.5
